# Structural, Electronic Properties, and Relative Stability Studies of Low-Energy Indium Oxide Polytypes Using First-Principles Calculations

**DOI:** 10.1021/acsomega.3c00105

**Published:** 2023-03-30

**Authors:** Arthi Devamanoharan, Vasu Veerapandy, Ponniah Vajeeston

**Affiliations:** † Department of Computational Physics, School of Physics, Madurai Kamaraj University, Madurai 625021, India; ‡ Department of Chemistry, Center for Materials Science and Nanotechnology, University of Oslo, Oslo 0371, Norway

## Abstract

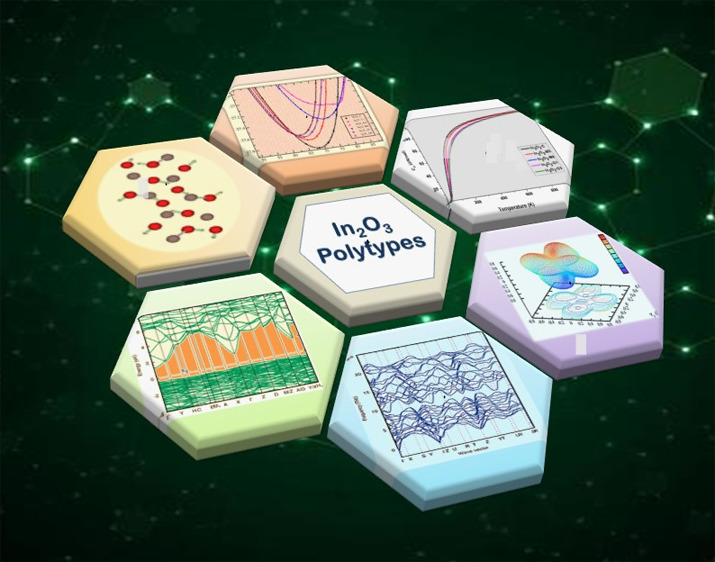

Materials made of indium oxide (In_2_O_3_) are now being used as a potential component of the next generation of computers and communication devices. Density functional theory is used to analyze the physical, electrical, and thermodynamical features of 12 low-energy bulk In_2_O_3_ polytypes. The cubic structure In_2_O_3_ is majorly used for many of the In_2_O_3_-based transparent conducting oxides. The objective of this study is to explore other new stable In_2_O_3_ polytypes that may exist. The structural properties and stability studies are performed using the Vienna ab initio simulation package code. All the In_2_O_3_ polytypes have semiconductive properties, according to electronic band structure investigations. The full elastic tensors and elastic moduli of all polytypes at 0 K are computed. Poisson’s and Pugh’s ratio confirms that all stable polytypes are ductile. The phonon and thermal properties including heat capacity are obtained for mechanically stable polytypes. For the first time, we report the Raman and infrared active modes of stable polytypes.

## 1 Introduction

Metal oxide semiconductors (MOSs) have received a lot of attention from materials scientists in recent years because of their numerous applications in a variety of industries, including electronics, catalysis, energy storage and conversion, adsorption, optoelectronics, and sensing. These features include a high surface-to-volume ratio, the ability to harvest light, surface permeability, and electrochemical and photochemical properties.^1,2^ Indium oxide (In_2_O_3_) has received the most attention among the many MOSs due to its good optoelectronic capabilities, stability, high electrical conductivity, and wide band gap.^3^

At ambient temperature, In_2_O_3_ is an *n*-type semiconductor with a direct band gap energy of 3.6 eV.^4^ The crystalline form exists in cubic (bixbyite type) and rhombohedral (corundum type) forms. Although pure In_2_O_3_ is rarely used in technical applications, it is the source of many transparent conducting oxide (TCO) and transparent oxide semiconductor (TOS) devices.^5^

They are now being explored as thin-film transistor (TFT) materials which is an enabling technology for the succeeding generation of computing and communication devices. It is well known that In_2_O_3_-based nanostructured thin films can act as an excellent *n*-type TCO as it has the property of transmitting light in the infrared (IR) and visible range of the electromagnetic spectrum.^6^ The low formation energy of bcc-Sn doped In_2_O_3_ suggests a larger abundance of both the neutral and cationic states of the Sn dopant.^7^ Indium tin oxide (ITO) is the most commonly used efficient TCO among the fabricated In_2_O_3_-based thin films because of its low energy of defect formation for enhancing better electrical characteristics.^7,8^ It is worth noting that the optical properties of In_2_O_3_-based thin films primarily depend upon postannealing temperature, film microstructure, film physical thickness, surface roughness, level of impurities, and deposition parameters.^5,7,8^ Various values as determined from the reported studies demonstrate that In_2_O_3_-based thin films show high optical transparency (82–93%) of the films.^9^ ITO thin films are one of the most extensively used TCOs in this regard. Moreover, surface-textured films can be applied for photocatalysis, photoelectrochemical applications, and light frequency modulation once the thin-film surface has been modified by periodic texturing and soft lithography.^1^ Recently, it has been used as a working electrode in electrochemical analysis because it offers electrochemical stability and a low background current. Recent research work by Silah et al. focused on the characteristics and sensor applications of modified ITO electrodes for the detection of various biomarkers, pathogens, pesticides, drugs, organic species, metals, etc.^10^

According to a recent study, a novel indium oxide modified with copper (Cu-In_2_O_3_) exhibits a record-breaking rate of CO_2_ conversion at relatively low temperatures (400–500 °C), making it the frontrunner among oxygen storage materials needed for low-temperature CO_2_ conversion, signifying a sustainable e-fuel.^11^ An experimental study by Zhao et al. explained that amorphous indium gallium zinc oxide TFTs and gas sensors are fabricated on flexible large-area substrates and offer an intriguing platform to develop wearable sensing devices due to their flexibility, conformability to the human body, and low cost. This gives excellent mechanical stability, electrical conductivity, and optical clarity to the material.^12^ The cubic-indium oxide-bixbyite structure is the crystalline form for various In_2_O_3_-based TCOs and TOSs.

Several crystal forms for a single chemical composition are known as polytypes, and they may be accessible when pressure and/or temperature are stimulated. Polytypes can give access to a variety of physical features in metal oxides that go beyond those of the most thermodynamically stable or common phases. The pressure-dependent phase transition studies of In_2_O_3_ have been reported in detail by Manjón et al.^13^ A high-pressure X-ray diffraction (XRD) study of both nanoparticle and bulk samples of In_2_O_3_ claimed cubic to rhombohedral phase transitions at room temperature when the sample is exposed to pressures between 12 and 25 GPa.^14^ Similarly, a combination of experimental and theoretical study by García-Domene et al. confirmed that bulk cubic In_2_O_3_ undergoes a phase transition to an orthorhombic phase (*Pbcn*) at pressures above 31 GPa at room temperature.^15^ Yusa et al. observed another phase transition from an orthorhombic phase (*Pbcn*) to another orthorhombic phase (*Pnma*) above 40 GPa and at 2000 K.^16^ A recent study on the La^3+^ doping process modified the size and morphology of the cubic In_2_O_3_ nanostructures into the rhombohedral In_2_O_3_ phase.^17^ Likewise, Sr^2+^ doping of cubic In_2_O_3_ nanowires causes phase transition into more stable rhombohedral In_2_O_3_ without high-temperature and high-pressure conditions.^18^

XRD, powder neutron diffraction, and Raman spectral analyses can all be used experimentally to determine the system’s crystal structure. On the other hand, there is no unique technique for theoretically identifying the ground state structure. First-principles calculations employing structural inputs from the inorganic crystal structure database (ICSD) were used to predict the equilibrium crystal structures, and the results mostly agree with experimental structures. The ICSD approach predicts the structural properties of hydrides and oxides accurately when more existing structural information (within similar chemical formulas; e.g., for the present case A_2_X_3_; A and X are elements in the periodic table) is used as a starting point. The reliability of the calculation depends upon the number of input structures considered in the calculations. The selection of input structures from 3035 entries for the A_2_X_3_ composition in the ICSD database is a tedious process, which also involves tremendous computations. Many phases share the same structural type, and in some instances, the positional parameters only differ slightly (for certain atoms). These structures mostly converted to a similar type of structural arrangement during the full geometry optimization even though we utilized different positional parameters, hence these possibilities are omitted. In this composition, almost 90 structure types have unique structural arrangements which are listed in Table S1 in the Supplementary Information.

As far as we are aware, no comprehensive property and stability studies of 12 In_2_O_3_ polytypes have been conducted in depth. In this article, the structural, electronic, mechanical, dynamic, and thermal features supported by the band structure, density of states, elastic constants, phonon dispersion with phonon density, and thermal parameters have all been investigated.

The rest of this paper has been organized as follows: In the results and discussion section, we cover a variety of topics under distinct subsections, including structural, electronic property, and mechanical stability study of 12 polytypes of In_2_O_3_. Then phonon studies of 10 mechanically stable In_2_O_3_ polytypes are investigated to find the dynamic stability. Finally, the thermal properties and Raman-IR study results of mechanically as well as dynamically stable In_2_O_3_ polytypes are reported. The computational methodology is briefly outlined in the next section. In conclusion, the important features of our calculations are summarized.

## 2 Results and Discussion

### 2.1 Structural Properties

Twelve low-energy In_2_O_3_ polytypes that were chosen are from five different crystal systems: one hexagonal (denoted as In_2_O_3_-*H*), one cubic (indicated as In_2_O_3_-*C*), two trigonal (as In_2_O_3_-*T*1, In_2_O_3_-*T*2), four monoclinic (namely, In_2_O_3_-*M*1, In_2_O_3_-*M*2, In_2_O_3_-*M*3, In_2_O_3_-*M*4), and four orthorhombic (as In_2_O_3_-*O*1, In_2_O_3_-*O*2, In_2_O_3_-*O*3, In_2_O_3_-*O*4). A sequence of convergence tests, including exchange-correlation potentials, **k**-point set, and cut-off energy are carried out to obtain an optimum crystal structure. Lattice and positional parameters have been determined via structural optimization based on total energy calculations.

**Figure 1:**
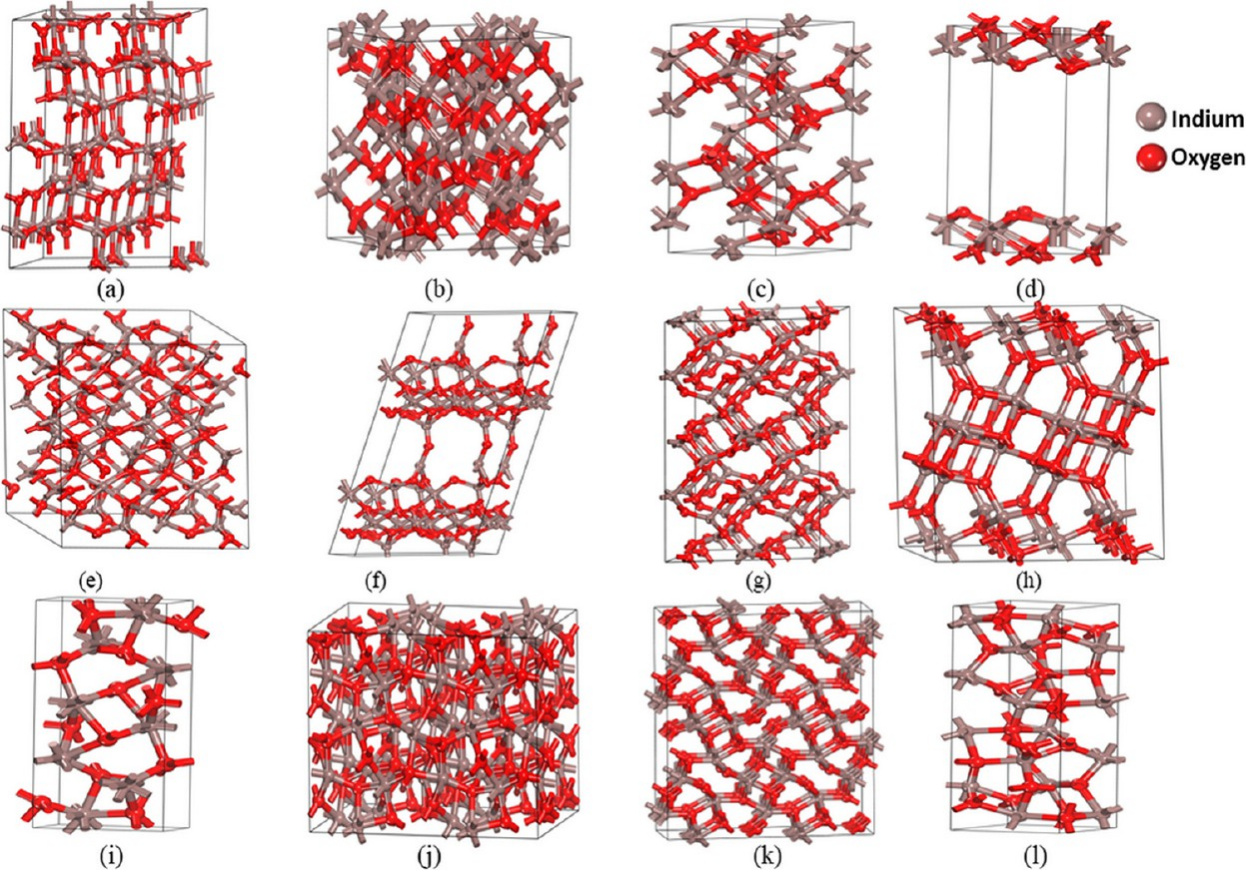
Optimized crystal structure of In_2_O_3_ polytypes: (a) In_2_O_3_-*H*, (b) In_2_O_3_-*C*, (c) In_2_O_3_-*T*1, (d) In_2_O_3_-*T*2, (e) In_2_O_3_-*M*1, (f) In_2_O_3_-*M*2, (g) In_2_O_3_-*M*3, (h) In_2_O_3_-*M*4, (i) In_2_O_3_-*O*1, (j) In_2_O_3_-*O*2, (k) In_2_O_3_-*O*3, and (l) In_2_O_3_-*O*4.

The crystal structures are schematically presented in Figure 1. The calculated lattice parameters and the Wyckoff positions of In_2_O_3_ polytypes are provided in Table 1. The computed lattice constants correlate well with the available experimental data, demonstrating the validity of the outcomes produced by the present density functional theory (DFT) approaches.^19−23^ For the In_2_O_3_-*C* crystal system with space group *Ia*3̅, the calculated lattice parameters are about 1.6% larger than the experimental data, while for polytypes In_2_O_3_-*H*, In_2_O_3_-*T*1, In_2_O_3_-*O*2, and In_2_O_3_-*O*4 with space groups *P*6_1_, *R*3̅*c*, *Pbcn*, and *Pbca*, the obtained lattice constants are slightly smaller than the reported values.^15^ Trigonal polytype In_2_O_3_-*T*2 has the highest unit cell volume (*V* = 428.07 Å^3^) than other systems.

**Table 1:** Optimized Equilibrium Lattice Parameters and Positional Parameters of In_2_O_3_ Polytypes with the Available Experimental Values

polytype name with space group	unit cell constants	atom	site	coordinates		
				*x*	*y*	*z*
**In_2_O_3_-*H***	*a* = 5.37 Å	In (1)	6a	0.2934	0.9933	0.1401
*P*6_1_ (No.169)	*b* = 5.37 Å	In (2)	6a	0.6591	0.6679	0.1742
[ICSD:001376]	*c* = 14.42 Å	O (1)	6a	0.6147	0.9531	0.1268
		O (2)	6a	0.9396	0.7062	0.1025
		O (3)	6a	0.3299	0.3311	0.1774
**In_2_O_3_-*C***	*a* = 10.29 Å; 10.12Å;^19^ 10.13Å;^21^ 10.09Å^20^	In (1)	24d	0.5335	0.5000	0.2500
*Ia*3̅ (No. 206)	*b* = 10.29 Å; 10.12Å;^19^ 10.13Å;^21^ 10.09Å^20^	In (2)	8b	0.7500	0.2500	0.2500
[mp-22598]	*c* = 10.29 Å; 10.12Å;^19^ 10.13Å;^21^ 10.09Å^20^	O (1)	48e	0.3821	0.3899	0.1542
**In_2_O_3_-*T*1**	*a = b* = 5.36 Å; 5.48Å;^20^ 5.49 Å^21^	In (1)	4c	0.3618	0.3618	0.3618
*R*3̅*c* (No. 167) [ICSD:009646]	*c* = 14.46 Å; 14.51Å;^20^ 14.53 Å^21^	O (1)	6e	0.2500	0.5381	0.9619
**In_2_O_3_-*T*2**	*a = b* = 5.75 Å	In (1)	2c	0.0000	0.0000	0.1016
*P*_321_ (No. 150)	*c* = 14.95 Å	In (2)	2d	0.3333	0.6667	0.0860
[mp-985587]		In (3)	2d	0.3333	0.6667	0.8748
		O (1)	6g	0.0170	0.6746	0.1572
		O (2)	3e	0.0000	0.3252	0.0000
**In_2_O_3_-*M*1**	*a* = 6.3032 Å	In (1)	4e	0.6288	0.1391	0.9466
*P* 2_1_/*c* (No. 14)	*b* = 6.7428 Å	In (2)	4e	0.1829	0.3288	0.0598
[ICSD:061089]	*c* = 16.2234 Å	In (3)	4e	0.0605	0.3841	0.3180
		In (4)	4e	0.4808	0.8983	0.7574
		O (1)	4e	0.8389	0.3694	0.9972
		O (2)	4e	0.4702	0.3717	0.1615
		O (3)	4e	0.4025	0.1792	0.8229
		O (4)	4e	0.8850	0.1215	0.2674
		O (5)	4e	0.1900	0.4685	0.6372
		O (6)	4e	0.2965	0.1253	0.9743
**In_2_O_3_-*M*2**	*a* = 11.3106 Å	In (1)	4b	0.4991	0.2525	0.7508
*Cm* (No. 8)	*b* = 6.4699 Å	In (2)	2a	0.8424	0.0000	0.2654
[mp-684944]	*c* = 11.8800 Å	In (3)	2a	0.7952	0.0000	0.9340
		In (4)	2a	0.8035	0.5000	0.4490
		In (5)	2a	0.7435	0.5000	0.7481
		In (6)	2a	0.7274	0.0000	0.6626
		In (7)	2a	0.5090	0.5000	0.5021
		O (1)	4b	0.8412	0.2221	0.8212
		O (2)	4b	0.8724	0.2253	0.3925
		O (3)	4b	0.6198	0.2686	0.6278
		O (4)	2a	0.8997	0.0000	0.6288
		O (5)	2a	0.8807	0.5000	0.6422
		O (6)	2a	0.8592	0.0000	0.1056
		O (7)	2a	0.6088	0.5000	0.8431
		O (8)	2a	0.6173	0.5000	0.3878
		O (9)	2a	0.6110	0.0000	0.8351
**In_2_O_3_-*M*3**	*a* = 6.5574 Å	In (1)	4e	0.9508	0.5390	0.7279
*P*2_1_/*c* (No. 14)	*b* = 9.2746 Å	In (2)	4e	0.5261	0.6603	0.2321
[mp-754531]	*c* = 6.6691 Å	O (1)	4e	0.8261	0.1040	0.4056
		O (2)	4e	0.7636	0.0167	0.8427
		O (3)	4e	0.6776	0.7063	0.5886
**In_2_O_3_-*M*4**	*a* = 4.0152 Å	In (1)	4e	0.8426	0.0527	0.7166
*P*2_1_/*c* (No. 14)	*b* = 13.4489 Å	In (2)	4e	0.6119	0.1637	0.1919
[mp-755066]	*c* = 6.1568 Å	O (1)	4e	0.9459	0.6097	0.4346
		O (2)	4e	0.6780	0.4940	0.8257
		O (3)	4e	0.6547	0.1950	0.5434
**In_2_O_3_-*O*1**	*a* = 5.3779 Å	In (1)	4c	0.1648	0.2500	0.1947
*Pnma* (No. 62)	*b* = 2.9762 Å	In (2)	4c	0.2356	0.7500	0.4503
[mp-644741]	*c* = 12.3614 Å	O (1)	4c	0.0136	0.2500	0.3931
		O (2)	4c	0.1305	0.7500	0.0637
		O (3)	4c	0.1333	0.2500	0.7791
**In_2_O_3_-*O*2**	*a* = 7.34 Å; 7.96 Å;^22^ 7.92 Å^23^	In (1)	8d	0.1239	0.2431	0.5346
*Pbcn* (No. 60)	*b* = 5.08 Å; 5.48 Å;^22^ 5.48 Å^23^	O (1)	8d	0.1448	0.3913	0.8948
[mp-1105681]	*c* = 5.00 Å; 5.59 Å;^22^ 5.59 Å^23^	O (2)	4c	0.0000	0.0520	0.2500
**In_2_O_3_-*O*3**	*a* = 7.9283 Å	In (1)	4c	0.0131	0.7500	0.8099
*Pnma* (No. 62)	*b* = 2.9507 Å	In (2)	4c	0.1924	0.7500	0.4917
[mp-1105699]	*c* = 8.2504 Å	O (1)	4c	0.0522	0.2500	0.6253
		O (2)	4c	0.1168	0.7500	0.0572
		O (3)	4c	0.2243	0.2500	0.3053
**In_2_O_3_-*O*4**	*a* = 5.247 Å; 5.52 Å^15^	In (1)	8c	0.2040	0.8119	0.0192
*Pbca* (No. 61)	*b =* 14.669 Å; 15.51 Å^15^	In (2)	8c	0.7375	0.4340	0.9932
[mp-1194571]	*c* = 5.078 Å; 5.38 Å^15^	O (1)	8c	0.1389	0.1991	0.1581
		O (2)	8c	0.6437	0.5551	0.1301
		O (3)	8c	0.4887	0.8772	0.1999

The In_2_O_3_-*H* polytype crystallizes in the hexagonal *P*6_1_ space group, comprising two types of indium (In) atoms and two types of oxygen (O) atoms located at the 6a site. The In_2_O_3_-*C* polytype comprises two types of indium atoms (they are surrounded by oxygen in the octahedral and trigonal prismatic coordination) and one type of oxygen atom fixed at Wyckoff positions 8b, 24d, and 48e, respectively. In_2_O_3_-*T*1 is corundum structured and crystallizes in the trigonal *R*3̅*c* space group. This polytype consists of one type of indium and one type of oxygen atom occupying 4c and 6e sites, respectively. In the first indium site, In (1) is bonded to six O (1) atoms to form a mixture of distorted edge, corner, and face-sharing InO_6_ pentagonal pyramids. In the first oxygen site, O (1) is bonded in a distorted trigonal pyramidal geometry to four In (1) atoms.

In_2_O_3_-*T*2 crystallizes in the trigonal space group *P*321. This is a two-dimensional structure, which contains one In_2_O_3_ sheet aligned in the (0,0,1) direction. There are three inequivalent indium sites. In the first indium site, In (1) is bonded to three O (1) and three O (2) atoms to form a mixture of face, corner, and edge-sharing InO_6_ octahedra. In the second indium site, In (2) is bonded to three O (1) and three O (2) atoms to form a mixture of corner and edge-sharing InO_6_ octahedra. In the third indium site, In (3) is bonded in a distorted trigonal noncoplanar geometry to three O (1) atoms. There are two inequivalent oxygen sites. In the first oxygen site, O (1) is bonded in a trigonal noncoplanar geometry to one In (1), one In (2), and one In (3) atom. In the second oxygen site, O (2) is bonded in a 4-coordinate geometry to two In (1) and two In (2) atoms.

Among the monoclinic polytypes, In_2_O_3_-*M*3 and In_2_O_3_-*M*4 crystallize in the monoclinic *P*2_*1*_/*c* space group. They consist of two inequivalent indium types and three types of oxygen atoms in the 4*e* site. In the first indium site, In (1) is bonded to one O (3), two O (1), and two O (2) atoms to form InO_5_ trigonal bipyramids. In the second indium site, In (2) is bonded to one O (1), one O (2), and two O (3) atoms to form distorted InO_4_ tetrahedra that share corners with two In(2)O_4_ tetrahedra, corners with four In(1)O_5_ trigonal bipyramids. In the first oxygen site, O (1) is bonded in a trigonal planar geometry to one In (2) and two In (1) atoms. In the second oxygen site, O (2) is bonded in a distorted trigonal noncoplanar geometry to one In (2) and two In (1) atoms. In the third oxygen site, O (3) is bonded in a distorted trigonal planar geometry to one In (1) and two In (2) atoms. Alternatively, In_2_O_3_-*M*1 has four types of indium atoms and six types of oxygen atoms situated in the same 4e site.

In_2_O_3_-*O*1 and In_2_O_3_-*O*3 among four orthorhombic polytypes are stibnite structured and crystallize in the orthorhombic *Pnma* space group, consisting of two types of indium and three types of oxygen atoms located at Wyckoff positions 4c site. In the first indium site, In (1) is bonded to two O (1), two O (3), and three O (2) atoms to form a mixture of distorted corner and edge-sharing InO_7_ pentagonal bipyramids. In the second indium site, In (2) is bonded in a seven-coordinate geometry to two equivalent O (2), two equivalent O (3), and three O (1) atoms. In the first oxygen site, O (1) is bonded in a distorted trigonal bipyramidal geometry to two In (1) and three In (2) atoms. In the second oxygen site, O (2) is bonded in a distorted trigonal bipyramidal geometry to two In (2) and three In (1) atoms. In the third oxygen site, O (3) is coupled to two In (1) and two In (2) atoms in a deformed rectangular seesaw-like shape.

In_2_O_3_-*O*4 crystallizes in the orthorhombic *Pbca* space group and has a corundum-like structure. There are two inequivalent types of indium atom and three inequivalent oxygen atom types at the 8*c* site. In the first indium site, In (1) is bonded to one O (2), two O (3), and three O (1) atoms to form distorted corner-sharing InO_6_ pentagonal pyramids. In the second indium site, In (2) is bonded in a six-coordinate geometry to one O (1), two O (3), and three O (2) atoms. In the first oxygen site, O (3) is bonded in a distorted trigonal pyramidal geometry to two In (1) and two In (2) atoms. In the second oxygen site, O (1) is bonded in a distorted tetrahedral geometry to one In (2) and three In (1) atoms. In the third oxygen site, O (2) is bonded in a distorted tetrahedral geometry to one In(1)and three In (2) atoms. In_2_O_3_-*O*2 is corundum-like structured and crystallizes in the orthorhombic *Pbcn* space group. There is one type of indium atom fixed at the 8d site and two types of inequivalent oxygen atoms at the 8d and 4c sites, respectively. In the first indium site, a mixture of the distorted corner, edge, and face-sharing InO_6_ octahedra is formed by the bonding of In (1) to two O (2) and four O (1) atoms. In the second oxygen site, O (2) is bonded in a distorted trigonal pyramidal geometry to four equivalent In (1) atoms.

**Figure 2:**
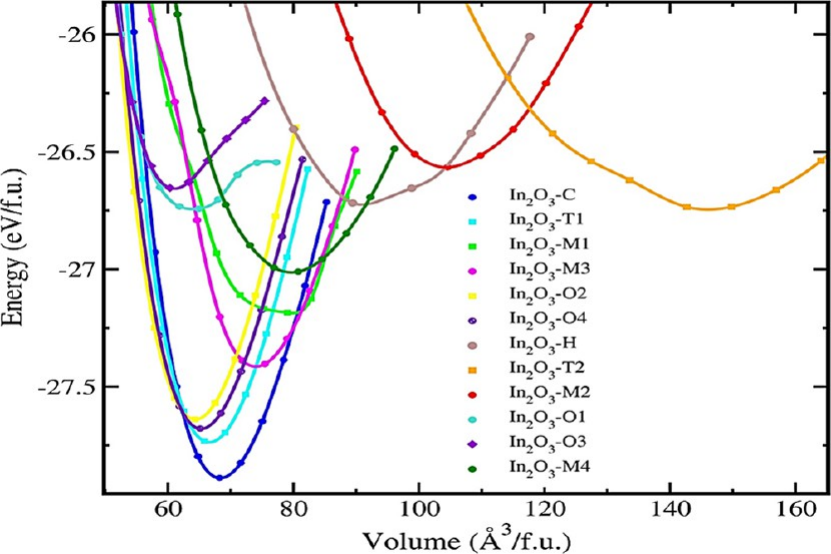
Calculated total energy as a function of the volume for 12 low-energy In_2_O_3_ polytypes. All the energy volumes are standardized to one formula unit (f.u.).

The data are fitted to the Birch–Murnaghan equation of state after the energy–volume curves for each polytype are examined, allowing for full relaxation at each volume.^24^ The total energy per unit cell is plotted against the volume to achieve the optimal structure (Figure 2).

**Figure 3:**
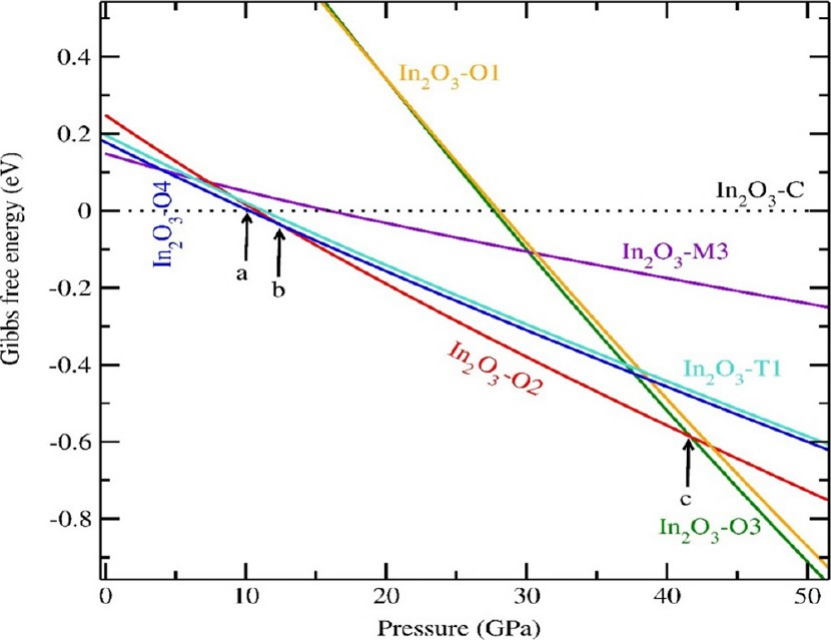
Calculated pressure versus Gibbs free energy plot for selected In_2_O_3_ polytypes. In_2_O_3_-*C* is the reference structure. Pressure involved in the phase transition from In_2_O_3_-*C* to In_2_O_3_-*O*4 is 10.1 GPa (at point *a*), from In_2_O_3_-*O*4 to In_2_O_3_-*O*2 is 12.9 GPa (at point *b*), and from In_2_O_3_-*O*2 to In_2_O_3_-*O*3 is 41.7 GPa (at point *c*).

It is possible to calculate the equilibrium energy, equilibrium volume, equilibrium bulk modulus, and its derivative by fitting the energy–volume curve. The minimum energy of the polytypes ranges from −27.9 to −26.5 eV/f.u. Additionally, the equilibrium volume of polytypes In_2_O_3_-*T*1, In_2_O_3_-*O*1, In_2_O_3_-*O*2, and In_2_O_3_-*O*4 are closer to the equilibrium volume of In_2_O_3_-*C*. From Figure 2, In_2_O_3_-*O*4 (−27.7 eV/f.u.) is found to be energetically stable next to the most stable polytype In_2_O_3_-*C.* In_2_O_3_-*M*2 and In_2_O_3_-*T*2 have higher total energies of above −26.8 eV/f.u. and have equilibrium volumes higher than 100 Å^3^/f.u. which indicates that they are energetically less stable than other polytypes. It is clear from Figure 2, that at lower volumes and higher-pressure conditions, In_2_O_3_-*C* can be transformed into other crystal systems with space groups *R*3̅*c*, *Pbcn*, *Pnma*, and *Pbca*. To find the possible phase transitions, we have plotted pressure versus Gibbs free energy, as shown in Figure 3. The phase transition from In_2_O_3_-*C* to In_2_O_3_-*T*1 is between 10 and 13 GPa, which agrees with the experimental result reported by Liu et al.^14^ Also In_2_O_3_-*C* is transformed into In_2_O_3_-*O*4 at 10.1 GPa, and In_2_O_3_-*O*4 is transformed into In_2_O_3_-*O*2 at 12.9 GPa, and In_2_O_3_-*O*2 is transformed into In_2_O_3_-*O*3 at 41.7 GPa.^16^

Formation energy (Δ*H*) is one of the significant properties of a compound that is directly related to its stability. It is the energy expended or needed in forming the compound from its constituent elements.^25^ We have determined the structural ground state parameters and calculated the formation energy for each In_2_O_3_ polytype [Table 2]. The Δ*H* is expressed below,
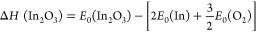
1

**Table 2:** List of In_2_O_3_ Polytypes with Minimum Energy (eV/f.u.) at their Equilibrium Volume (Å^3^/f.u.) and their Corresponding Formation Energy (eV/f.u.)

polytype name	In_2_O_3_-*H*	In_2_O_3_-*C*	In_2_O_3_-*T*1	In_2_O_3_-*T*2	In_2_O_3_-*M*1	In_2_O_3_-*M*2	In_2_O_3_-*M*3	In_2_O_3_-*M*4	In_2_O_3_*-O*1	In_2_O_3_-*O*2	In_2_O_3_-*O*3	In_2_O_3_-*O*4
minimum energy *E*_0_ (eV/f.u.)	–26.717	–27.887	–27.732	–26.735	–27.184	–26.566	–27.010	–27.403	–26.742	–27.639	–26.654	–27.678
equilibriumvolume (Å^3^/f.u.)	89.44	68.22	65.81	142.69	79.04	104.56	80.74	75.44	64.92	64.29	60.32	65.14
formation energy (eV/f.u.)	–6.72	–7.89 [−7.81]^27^	–7.74 [−7.67]^27^	–6.74	–7.19	–6.57	–7.02	–7.41	–6.75	–7.65	–6.66	–7.68

where *E*_0_(In_2_O_3_) is the total energy of the compound per formula. *E*_0_(In) and *E*_0_(O_2_) are the total energies of indium and O_2_ molecule, respectively. For the formation energy calculation, indium is considered as bulk [mp-1,184,502, *R*3̅*c*] and the O_2_ molecule is placed in the center of a cubic box with lattice parameter 25 Å. For all polytypes, the value of Δ*H* is negative, indicating that they are stable.^26^ The formation energy of cubic (*Ia*3̅) and corundum (*R*3̅*c*) type In_2_O_3_ from the theoretical study by Tanaka et al. is close to the value found in this paper.^27^ This also confirms that In_2_O_3_-*C* (−7.89 eV/f.u.) is the most stable and In_2_O_3_-*M*2 (−6.57 eV/f.u.) is the least stable polytype.

### 2.2 Electronic Properties

**Figure 4:**
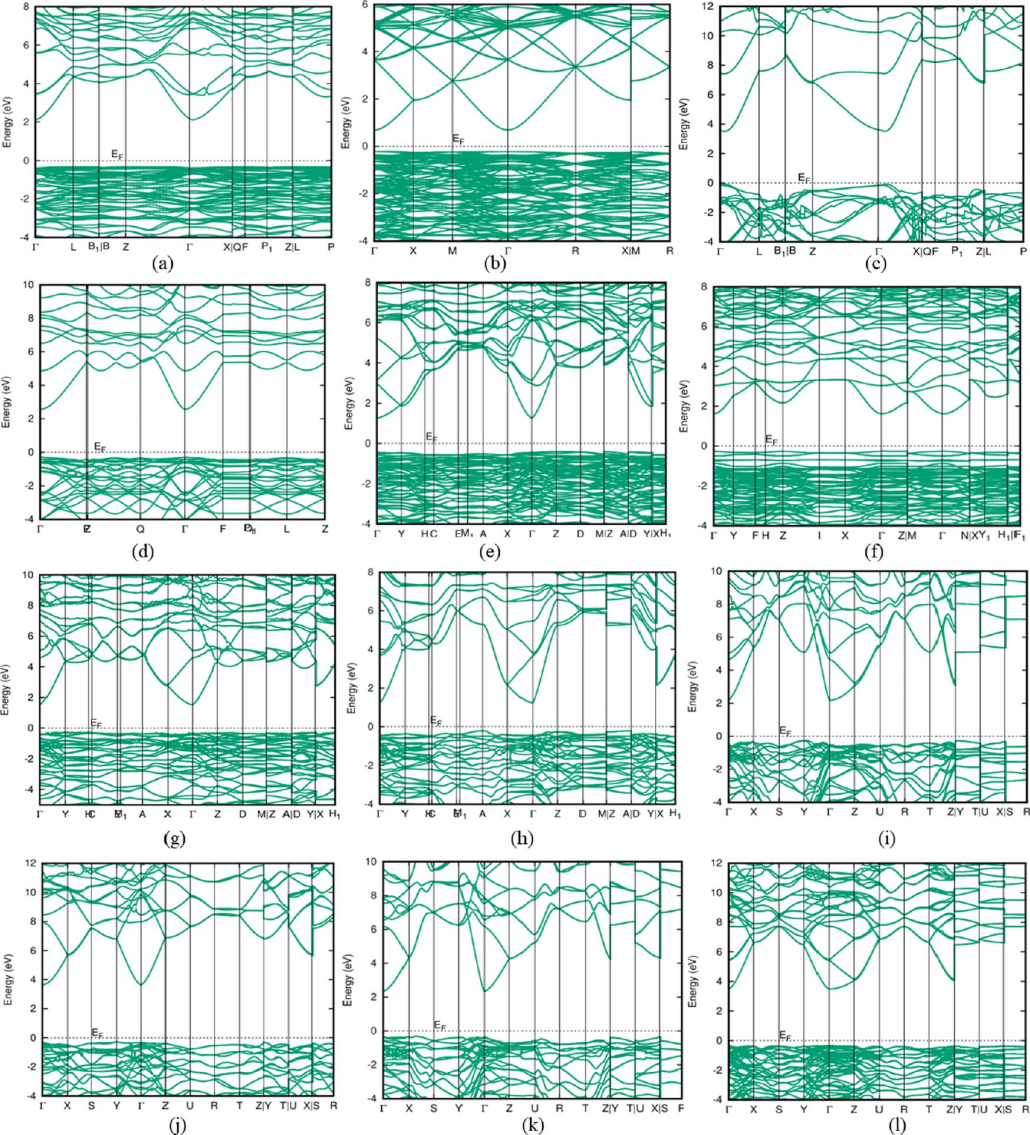
Computed band structures with the direct band gap of polytypes (a) In_2_O_3_-*H*, (b) In_2_O_3_-*C* (calculated using GGA approximation), (c) In_2_O_3_-*T*1, (d) In_2_O_3_-*T*2, (e) In_2_O_3_-*M*1, (f) In_2_O_3_-*M*2, (g) In_2_O_3_-*M*3, (j) In_2_O_3_-*O*2, (k) In_2_O_3_-*O*3, and (l) In_2_O_3_-*O*4 and with the indirect band gap of polytypes (h) In_2_O_3_-*M*4 and (i) In_2_O_3_-*O*1 calculated using hybrid DFT (HSE-06 level).

It is extremely desirable to study electronic properties to determine the potential outcomes from an application-based perspective.^28^ The value of the energy gap lays the way for modifying the required physical properties to meet the demands of modern technology.^28^ The fundamental concepts of band profiles, the total density of states, and partial densities of states are utilized to describe the electronic structure of the current In_2_O_3_ polytypes. The band structures are subsequently calculated at the theoretical equilibrium lattice constant. To find the feasible polytypes for photocatalytic processes, light-emitting diodes, solar cells, and electronics, detailed electronic computations are performed.^29^ DFT calculations on semiconductors are severely hampered by the band gap problem.^25,30^ The HSE-06 functional provides an accurate description of electronic structure for semiconducting materials.^31^ Our band structure calculations at the HSE-06 level show that all polytypes with an energy gap between 1.4 and 3.9 eV, are presented in Figure 4. The lowest conduction bands of all In_2_O_3_ polytypes arise from 5s orbitals of indium and the highest valence bands result from 2p orbitals of oxygen.^32^ The Fermi energy (*E*_F_) of the corresponding polytypes is set to be at the top of the valence band. The valence band maximum of all In_2_O_3_ polytypes lies in the range between −0.35 and −0.09 eV. The unit cell of In_2_O_3_-*C* contains 80 atoms and that requires more computation time to run the HSE-06 calculation. Hence in this study, only for the polytype In_2_O_3_-*C*, the band structure calculations were performed using the generalized gradient approximation (GGA). The calculated GGA band gap is about 0.93 eV (as listed in Table 3) compared with the reported value of 0.94 eV (GGA).^25^

**Table 3:** Computed Band Gap Values of In_2_O_3_ Polytypes

polytype name	In_2_O_3_-*H*	In_2_O_3_-*C*	In_2_O_3_-*T*1	In_2_O_3_-*T*2	In_2_O_3_-*M*1	In_2_O_3_-*M*2	In_2_O_3_-*M*3	In_2_O_3_-*M*4	In_2_O_3_-*O*1	In_2_O_3_-*O*2	In_2_O_3_-*O*3	In_2_O_3_-*O*4
space group	*P*6_1_	*Ia*3̅	*R*3̅*c*	*P*321	*P*2_1_*/c*	*Cm*	*P*2_1_*/c*	*P*2_1_*/c*	*Pnma*	*Pbcn*	*Pnma*	*Pbca*
band gap (eV)	2.56	0.91 [0.94]^25^	3.59 [3.40]^31^	2.87	1.76	1.92	1.89	1.45	2.40	3.94	2.63	3.94
band gap type	direct	direct	direct	direct	direct	direct	direct	indirect	indirect	direct	direct	direct

From the calculated energy band structures (HSE-06 level), the polytype In_2_O_3_-*M*4 is denoted to have a lower energy band gap (1.5 eV) compared to others. Polytypes with wider band gaps are In_2_O_3_-*O*3 (2.6 eV), and In_2_O_3_-*T*2 (2.9 eV). The calculated band gap value of polytype In_2_O_3_-*T*1 is 3.6 eV, which is 5.6% higher than the reported band gap value (3.40 eV).^31^ Two orthorhombic polytypes In_2_O_3_-*O*2 and In_2_O_3_-*O*4 have very high band gaps (nearly 4 eV). The direct gap at the Γ point (where *k* = 0,0,0) is found for In_2_O_3_-*H*, In_2_O_3_-*C*, In_2_O_3_-*T*1 and -*T*2, In_2_O_3_-*M*1, -*M*2, and -*M*3, and In_2_O_3_-*O*2, -*O*3, and -*O*4 which can be used as a material for optoelectronic devices. In_2_O_3_-*O*1 and In_2_O_3_-*M*4 polytypes have a small indirect band gap; since the conduction-band minimum is located at the high symmetrical point, the indirect band gap corresponds to an off-valence band maximum, which might result from the mixing of indium 4d and oxygen 2p states away from the zone center. The topmost valence band is flat, while the bottom-most conduction band of In_2_O_3_ polytypes is dispersive and located at the Γ point, which are the significant properties of TCO materials.^32^

### 2.3 Mechanical Stability

The elastic properties of solids deliver the relation between the mechanical and dynamic behavior of the crystal. The elastic constants bring a tool to obtain mechanical characteristics such as hardness, strength, shear modulus, Young’s modulus, bulk modulus, elastic stiffness coefficients, Poisson’s ratio, and melting temperature.^28^ They are also linked to the phonon density of states, phonon dispersion spectrum, heat capacity, entropy, and other thermodynamic parameters. A study by Ramzan et al. previously investigated the elastic characteristics of cubic In_2_O_3_.^33^ The other polytypes of In_2_O_3_ still require a complete theoretical knowledge of mechanical properties and these properties are studied for the first time in this study. It is important to emphasize that a comprehensive understanding of mechanical properties is required to unlock a material’s potential for various applications. The tensorial form of Hook’s law describes the linear dependency of the stress component *σ_i_*(*i* = 1 – 6) and the applied strain *ε_j_*(*j* = 1 – 6) under a minor deformation.
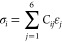
2

Here, *C_ij_* are the elastic constants of the crystal. eq 2 constitutes a set of six linear equations with 27 variables, namely, the 21 elastic constants and six components of stress (with *C_ij_* = *C_ji_*). In our study, solving the set of linear equations requires seven separate ab initio calculations with seven different levels of applied strain (−0.015, −0.010, −0.005, 0.0, 0.005, 0.010, and 0.015). The reliability of lattice elastic constants successfully predicts the stability and mechanical response of a material toward the external strain.^28^ The ability of the crystal to withstand the applied mechanical stress in the crystallographic directions *a*, *b*, and *c* is measured by the elastic constants *C*_11_, *C*_22_, and *C*_33_, respectively. For the In_2_O_3_-*C* polytype, it is noticed that *C*_11_ > *C*_12_ > *C*_44_. *C*_33_ is found to be smaller than *C*_11_ and *C*_22_ for In_2_O_3_-*M*1, -*M*2, -*O*1, -*O*3 and In_2_O_3_-*O*4, which indicates that the structure is more compressible in the *c*-direction and bonding within the plane-*ab* is much stronger than that extending in the out-of-plane directions. Likewise, *C*_11_ is smaller than *C*_33_ and *C*_22_ for In_2_O_3_-*M*4, and *C*_22_ is smaller for In_2_O_3_-*M*3 and In_2_O_3_-*O*2 which specifies that the structure is more compressible in the *a* and *b*-directions, respectively. The response of the crystal to shear is controlled by the elastic constants *C*_44_, *C*_55_, and *C*_66_ in all monoclinic and orthorhombic In_2_O_3_ polytypes. These elastic constants are particularly useful because the mechanical failure modes of crystalline solids are often controlled by shearing strain, rather than the uniaxial strains. *C*_44_ signifies the indentation hardness of materials. The small value of *C*_44_ in In_2_O_3_ -*M*1, -*M*2, -*M*3, -*O*1, -*O*2 indicates the material’s inability to resist the shear deformation in the (100) plane. The off-diagonal shear components of the elastic constants *C*_12_, *C*_13_, and *C*_23_ are due to the resistance to volume-conserving crystal distortion. All independent elastic constants of each structure obtained are operated to reproduce the various elastic parameters including bulk, Young’s, and shear moduli, which have been obtained by employing the Voigt–Ruess–Hill method, shown in Table 5. Elastic properties have been previously studied by Qi et al. for cubic-In_2_O_3_.^34^ For stable structures, the elastic constants need to meet the mechanical stability criterion. Born stability criteria are a set of conditions on the elastic constants (*C_ij_*), which are related to the second-order change in the internal energy of a crystal under deformation.^35^ Born stability criteria of the elastic constant for different polytypes are listed in Table 4.

**Table 4:** Crystal Systems with Their Corresponding Number of Independent Second-Order Elastic Constants *C_ij_* and Born Stability Criteria

crystal system	*C_ij_*	born stability criteria
hexagonal	5	*C*_11_ > *C*_12_; 2*C*_13_^2^ < *C*_33_(*C*_11_ + *C*_12_); *C*_44_ > 0
cubic	3	*C*_11_ – *C*_12_*>* 0; *C*_11_*+* 2*C*_12_*>* 0; *C*_44_*>* 0
trigonal	5	*C*_11_ – *C*_12_*>* 0; *C*_13_^2^*<* 0.5 *C*_33_ (*C*_11_*+ C*_12_); *C*_14_^2^*<* 0.5 *C*_44_ (*C*_11_*–C*_12_); *C*_44_*>* 0
orthorhombic	9	*C*_11_, *C*_44_, *C*_55_, *C*_66_*>* 0; *C*_11_*C*_22_*> C*_12_^2^; *C*_11_*C*_22_*C*_33_*+* 2*C*_12_*C*_13_*C*_23_ – *C*_11_*C*_23_^2^ – *C*_22_*C*_13_^2^ – *C*_33_*C*_12_^2^*>* 0
monoclinic	13	*C*_11_, *C*_22_, *C*_33_, *C*_44_, *C*_55_, *C*_66_ *>* 0; [*C*_11_ *+ C*_22_ *+ C*_33_ *+* 2(*C*_12_ *+ C*_13_ *+ C*_23_)] *>* 0; *C*_33_*C*_55_ – *C*_35_^2^ *>* 0*; C*_44_*C*_66_ – *C*_46_^2^ *>* 0; *C*_22_ *+ C*_33_ – 2*C*_23_ *>* 0

Born criteria of mechanical stability are obeyed by all the individual elastic constants of In_2_O_3_ polytypes with space group *P*6_1_, *Ia*3̅, *P*2_1_, *Pnma*, and *Pbca*, thereby predicting the stability of these materials. Polytypes In_2_O_3_-*T*2 and In_2_O_3_-*M*2 with space group *P*_321_ and *Cm* do not comply with the stability criteria and it is concluded that they are not mechanically stable.

**Figure 5:**
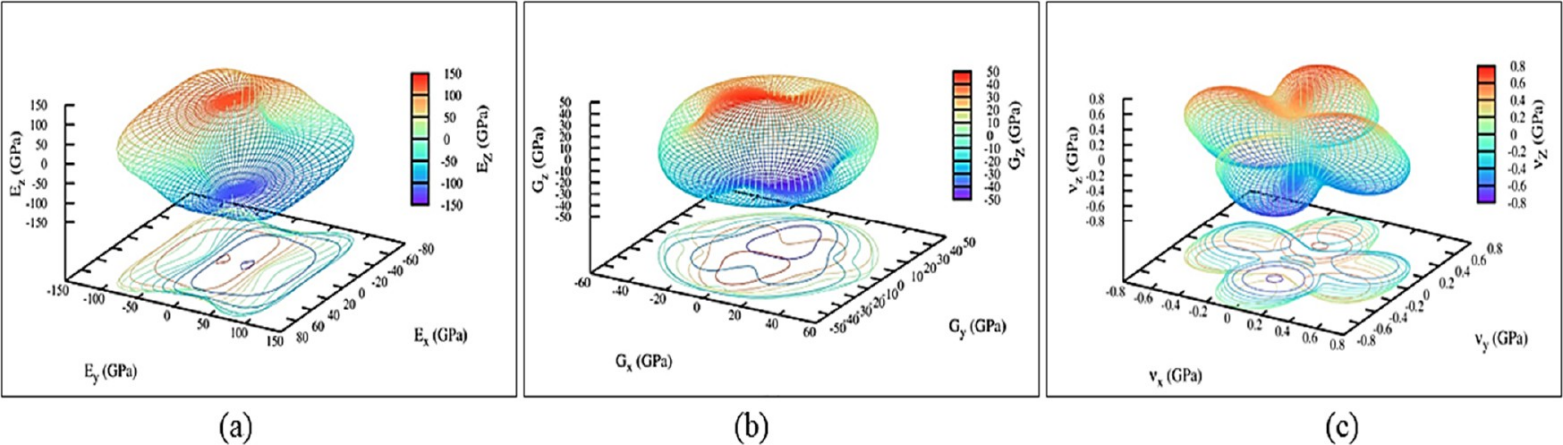
Spatial dependence of the (a) Young’s modulus, (b) shear modulus, and (c) Poisson’s ratio of the In_2_O_3_-M4 polytype. Directions *x*, *y*, and *z* represent the increments along the a, b, and c directions of the primitive cell shown in Figure 1

The Voigt approximation adopts a continuous strain distribution while allowing for stress discontinuities.^38^ Reuss approximation implies discontinuous strain distribution and constant stress instead.^39^ It is worth observing that Hill’s approximation takes the arithmetic average of these two limitations and, with suitable energy considerations, nearly reproduces the real situation in polycrystalline solids.^40^ The bulk modulus characterizes a compound’s resistance to plastic deformation and the shear modulus describes its resistance to a volume change as a result of isotropic pressure applied.^41^ The bulk modulus of polytype In_2_O_3_-*O*2 and In_2_O_3_-*O*3 with space group *Pbcn* and *Pnma,* respectively, is above 300 GPa, larger than those of other polytypes. The calculated bulk modulus values of polytypes In_2_O_3_-*M*1 and In_2_O_3_-*M*4 are in the range of 99 ± 32 Gpa of ITO films.^42^ Using the ELATE software, we show Young’s, shear modulus, and Poisson’s ratio of the In_2_O_3_-*M*4 polytype in Figure 5a–c, respectively.^43^ 3D spatial representations of elastic parameters for other polytypes are shown in Figures S2–S4 in the supplementary information.

Young’s modulus (*E*) calculates the resistance to longitudinal stress and evaluates the stiffness of solid compounds.^41^ The higher value of *E* is observed for In_2_O_3_-*O*1 (*E* = 258 GPa) and In_2_O_3_-*O*3 (*E* = 287 GPa) polytypes, which therefore have a more covalent nature than the remaining polytypes. The small value of *E* (<50 GPa) indicates that In_2_O_3_-*H* and In_2_O_3_-*T*1 cannot withstand large tensile stress. The Pugh’s ratio (*B*/*G*), which has a high value (>1.75), indicates ductility, whereas a low value (<1.75) indicates brittleness.^44^ Thus, all stable In_2_O_3_ polytypes are expected to show ductile features as their *B*/*G* values are higher than 1.75. Poisson’s ratio (ν) measures the stability of a crystal against shear and also predicts the failure mode of solids with a critical value of 0.26. If ν is greater (lesser) than 0.26, the material is ductile (brittle).^41^ The ν value of all mechanically stable In_2_O_3_ polytypes indicates that they are ductile in nature. The Poisson’s ratio in a pure covalent compound is about 0.10, whereas for metallic bonding, the value is about 0.33.^41^ This implies that metallic bonding occurs in all stable In_2_O_3_ polytypes.

### 2.4 Dynamic and Thermal Stability

**Figure 6:**
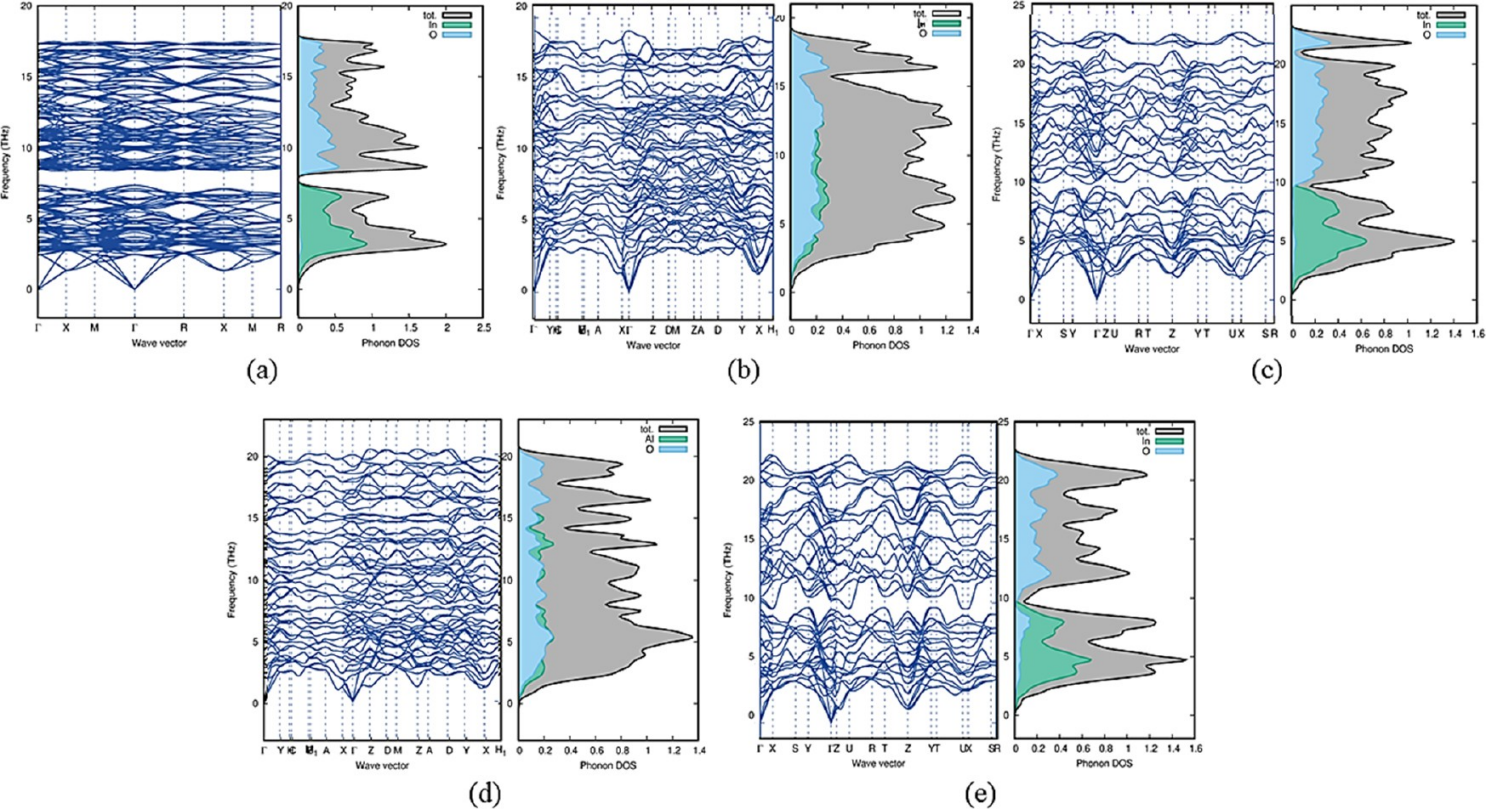
Computed phonon dispersion curve with PhDOS of mechanically stable In_2_O_3_ polytypes: (a) In_2_O_3_-*C*, (b) In_2_O_3_-*M*3, (c) In_2_O_3_-*M*4, (d) In_2_O_3_-*O*1, and (e) In_2_O_3_-*O*3 display positive modes.

The phonon dispersion relations are defined as the *k* dependency of the frequencies for all branches and chosen directions in the crystal.^45^ The phonon dispersion relations are drawn along the crystal high-symmetry axis of the Brillouin zone.^46^ For each polytype, phonon computations are performed to determine the dynamic stability. Phonon density of state (PhDOS) curves are calculated on a Monkhorst–Pack grid, and the dynamic matrices are obtained from the force constants.^47^ We estimated the phonon dispersion curves at the equilibrium volume along the high-symmetry direction for all the mechanically stable In_2_O_3_ polytypes in addition to the total phonon density of states and these variations are presented in Figures 6 and S1.^48^ Polytypes In_2_O_3_-*C*, In_2_O_3_-*M*3, In_2_O_3_-*M*4, In_2_O_3_-*O*1, and In_2_O_3_-*O*3 exhibit positive modes, which indicates that these polytypes are dynamically stable (Figure 6), whereas the polytypes In_2_O_3_-*H*, In_2_O_3_-*M*1, In_2_O_3_-*M*2, In_2_O_3_-*O*2, and In_2_O_3_-*O*4 show the presence of negative modes and indicate they are dynamically unstable (Figure S1).

From Figure 6, we conclude that for dynamically stable polytypes, higher frequencies above 8 THz are dominated by the smaller oxygen atom while lower frequencies are dominated by the heavier indium atom. However, for In_2_O_3_-*O*1, In_2_O_3_-*M*3, and In_2_O_3_-*M*4, few oxygen modes are present in the low-frequency region. Also, for the polytype In_2_O_3_-*M*4, a few indium modes are found above 10 THz.

A phonon band gap between optical and acoustic modes is found in mechanically stable polytypes In_2_O_3_-*C*, In_2_O_3_-*O*1, and In_2_O_3_-*O*3. This separation affects the phonon scattering processes and the lattice thermal conductivity. A large energy separation between the optical and acoustic modes found in polytype In_2_O_3_-*C* implies that the ionic bonds between the atoms are more rigid and the lattice thermal conductivity is relativity higher than in other polytypes. The small phonon bandgap found in In_2_O_3_-*M*3 and In_2_O_3_-*O*1 implies low lattice thermal conductivity and a high scattering process rate. Negative (imaginary) phonon frequencies found for polytypes In_2_O_3_-*H*, In_2_O_3_-*M*2, and In_2_O_3_-*O*4 at many wave vectors indicate dynamic instability. Such instability causes the entire lattice to undergo a structural change.

Solids have distinctive vibrational degrees of freedom due to the oscillatory motions of the constituent atoms. Several physical characteristics of materials, including specific heat, elastic constants, and melting temperature, are correlated with the Debye temperature. The Debye temperature, θ_D_, is defined as θ_D_ = ω_D_/*k*_B_, where *k*_B_ is Boltzmann’s constant and ω_D_ is the Debye frequency (highest frequency among the possible vibrational mode). The Debye temperature tends to increase with stronger atomic bonds and decreases with increasing atomic mass. Only acoustic modes produce vibrational excitations at low temperatures. As a result, the elastic constants are used to calculate θ_D_ at low temperatures.^49^ The calculated values of Debye temperature are listed in Table 5. According to the estimated elastic moduli, a high value of θ_D_ denotes the hardness of In_2_O_3_-*M*3, In_2_O_3_-*M*4, and In_2_O_3_-*O*3.

**Table 5:** Observed Values of Single-Crystal Elastic Constants *C_ij_* (GPa), Bulk Modulus *B* (GPa) Shear Modulus *G* (GPa), Poisson’s Ratio ν, Young’s Modulus *E* (GPa), Pugh’s Ratio (*B*/*G*), and Debye Temperature θ_D_ (K) of all In_2_O_3_ Polytypes^a^

polytype name	In_2_O_3_-*H*	In_2_O_3_-*C*	In_2_O_3_-*T*1	In_2_O_3_-*T*2	In_2_O_3_-*M*1	In_2_O_3_-*M*2	In_2_O_3_-*M*3	In_2_O_3_-*M*4	In_2_O_3_-*O*1	In_2_O_3_-*O*2	In_2_O_3_-*O*3	In_2_O_3_-*O*4
*C*_11_	45.4	221.2 [234.3]^36^	513.5	–149.1	131.5	73.1	111.2	120.0	464.3	454.6	552.1	406.7
*C*_12_	27.3	105.0 [107.2]^36^	480.9	–160.6	64.7	67.0	18.7	91.7	251.0	299.5	234.9	351.7
*C*_13_	29.4		332.6	11.6	33.9	59.6	43.2	69.3	234.5	386.7	312.5	264.9
*C*_14_	0.0		0.0	–3.5	0.0	0.0	0.0	0.0	0.0		0.0	
*C*_15_					8.2	–17.4	–2.5	11.5				
*C*_22_					125.2	118.8	27.9	144.9	530.6	422.6	545.4	425.8
*C*_23_					21.2	24.4	31.9	70.8	204.9	409.5	234.4	218.0
*C*_25_					3.9	–13.8	–3.3	–15.4				
*C*_33_	93.5		254.6	0.3	76.4	73.4	134.4	163.1	314.6	490.5	378.4	243.0
*C*_35_					–8.1	6.4	–8.2	–4.8				
*C*_44_	27.7	56.2 [62.7]^36^	8.1	–1.4	18.4	11.1	21.2	43.4	66.0	21.5	141.8	41.7
*C*_46_					17.5	–8.1	–8.2	–10.9				
*C*_55_					33.7	13.5	26.9	43.3	130.2	59.1	103.5	56.7
*C*_66_	9.1		16.3	5.8	44.8	28.3	21.3	38.8	97.7	65.4	81.4	18.5
*B*_V_	39.6	143.7	397.1	–63.6	63.7	63.0	51.2	99.1	298.9	395.5	337.7	319.4
*B*_R_	35.6	143.7	184.3	1.0	55.3	57.8	26.4	98.0	278.4	351.3	326.0	216.3
*B*_H_	37.6	143.7 [149.6]^36^	290.7 [212]^37^	–31.3	59.5	60.4	38.8	98.5	288.7	373.4 [210.6]^22^	331.9	267.8
*G*_V_	19.5	56.9	15.5	–10.1	33.6	18.2	25.9	38.2	100.1	48.9	111.6	35.1
*G*_R_	14.7	56.9	11.2	8.5	23.0	–406.1	18.6	29.8	91.3	34.0	99.7	26.5
*G*_H_	17.1	56.9 [63.0]^36^	13.4	–0.8	28.3	–193.9	22.2	34.0	95.7	41.0	105.7	30.8
ν_V_	0.3	0.3	0.5	0.4	0.3	0.4	0.3	0.3	0.3	0.4	0.4	0.4
ν_R_	0.3	0.3	0.5	–0.6	0.3	–2.1	0.2	0.4	0.4	0.5	0.3	0.4
ν_H_	0.3	0.3 [0.32]^36^	0.5	0.5	0.3	–22.5	0.3	0.3	0.4	0.4	0.4	0.4
*E*_V_	50.1	150.9	46.0	–28.8	85.7	49.8	66.4	101.5	270.1	138.2	301.6	101.6
*E*_R_	38.8	150.9	33.1	6.9	60.7	908.7	45.2	81.0	246.9	98.7	271.4	76.4
*E*_H_	44.5	150.9 [165]^36^	39.6	–2.3	59.5	8324.1	56.0	91.4	258.5	118.5	286.6	89.0
*B*/*G*_V_	2.0	2.5	25.6	6.3	1.9	3.5	2.0	2.6	3.0	8.3	3.0	9.1
*B*/*G*_R_	2.4	2.5	16.4	0.1	2.4	–0.1	1.4	3.3	3.0	10.3	3.3	8.2
*B*/*G*_H_	2.2	2.5 [2.37]^36^	21.7	40.0	2.1	–0.3	1.8	2.9	3.0	9.1	3.1	8.7
**θ**_D_	253.4	405.26	189.97	-	294.05	-	432.79	530.74	499.68	329.77	523.25	286.5

a Subscripts V, R, and H indicate the Voigt, Reuss, and Hill bounds correspondingly.

This paper describes the behavior of all dynamically stable polytypes of In_2_O_3_ in terms of thermodynamic properties such as temperature, free energy, entropy, and heat capacity. The temperature of the system increased from 0 to 1000 K for all stable In_2_O_3_ polytypes to calculate the thermal properties. Entropy is a measure of molecular disorder. When the temperature increases, entropy increases with a decrease in regularity. The entropy of polytype In_2_O_3_-*C* increased to 240 J/K/mol, whereas those of other dynamically stable polytypes increased to about 220 J/K/mol.

**Figure 7:**
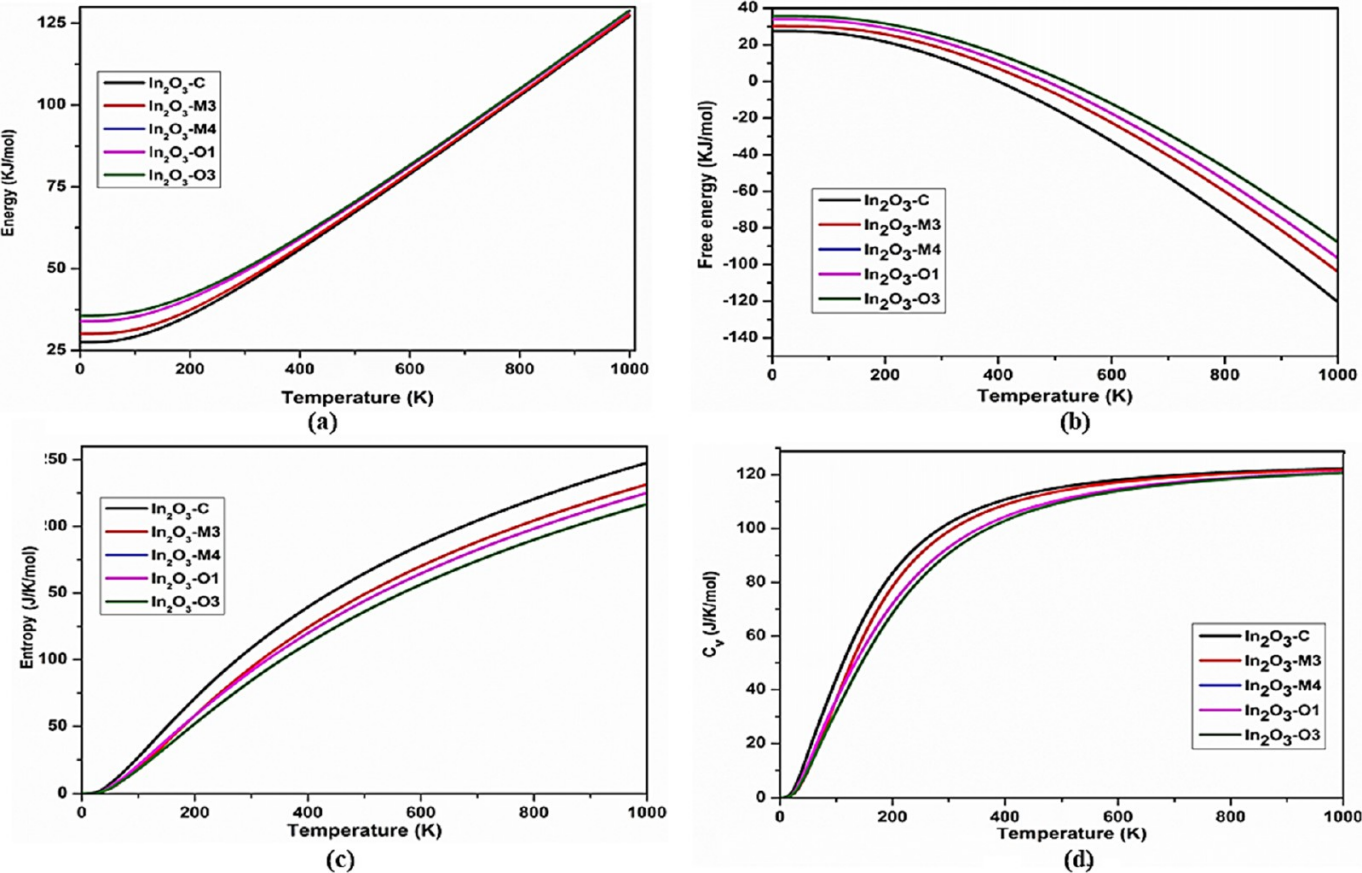
Thermal parameters, (a) internal energy, (b) free energy, (c) entropy, and (d) heat capacity as a function of temperature (K) for all mechanically and dynamically stable In_2_O_3_ polytypes.

Thermal capacity (*C*_v_) is the amount of heat required to make a unit change in its temperature. Figure 7d indicates all stable polytypes have high heat capacity (120 J/K/mol) and are more stable even for high temperatures.^50^ The internal energy of a system is found to increase with the increase in temperature. This increase in internal energy also depends on the amount of matter and found all stable polytypes have reached a very high internal energy of about 130 KJ/mol when the temperature of the system reaches 1000 K.

### 2.5 Raman and IR Studies

**Figure 8:**
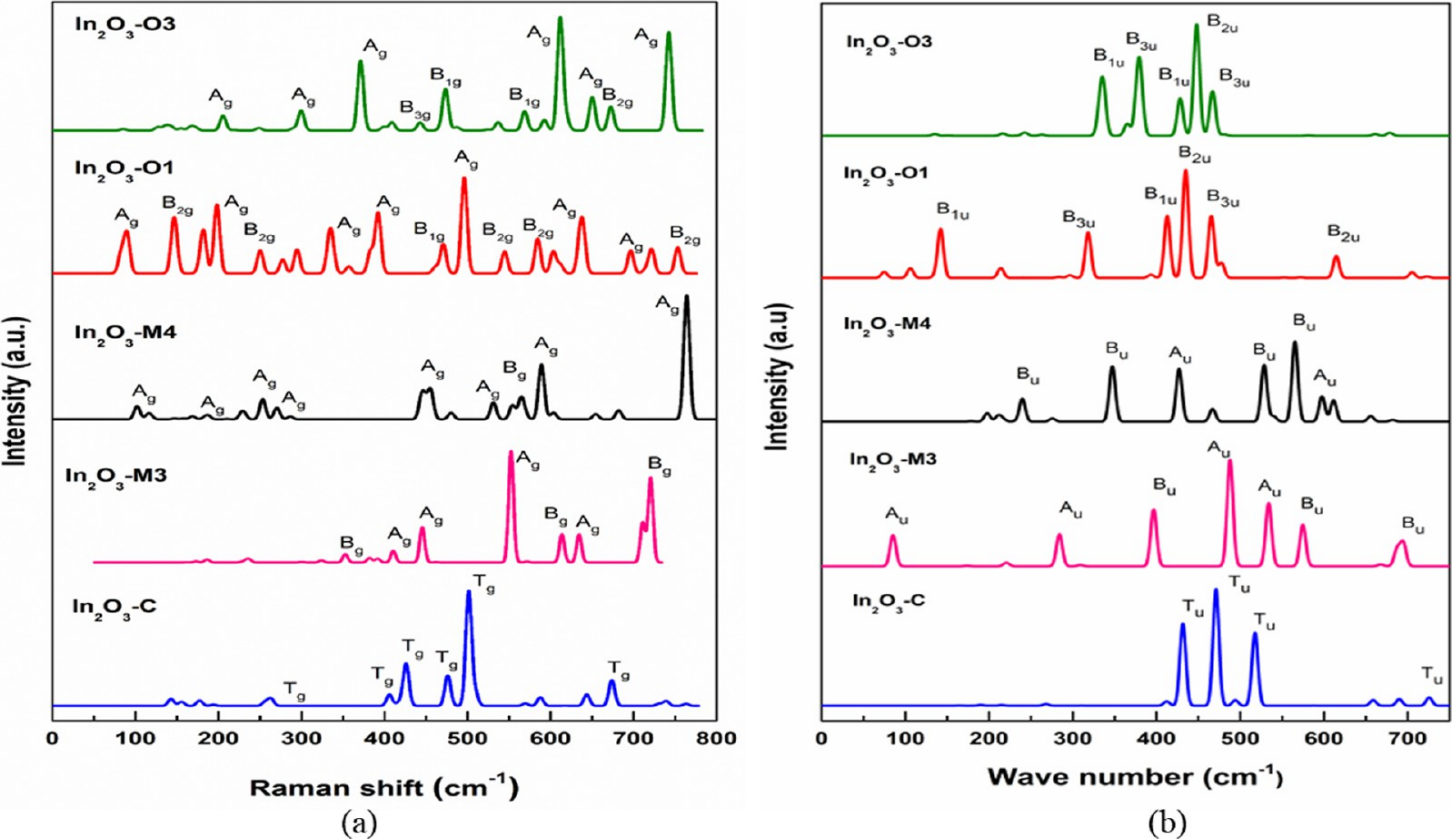
Calculated Raman (a) and IR (b) spectra for all mechanically and dynamically stable In_2_O_3_ polytypes.

Raman spectroscopy is a powerful technique for characterizing the zone center phonon properties in bulk crystals.^51^ The symmetry of the In_2_O_3_-*C* polytype can be described by the *Th*(*m-*3) point group and the irreducible representation of phonon modes at the center of the Brillouin zone is Γ = 4*A*_g_ + 4*E*_g_ + 14*T*_g_ + 5*A*_u_ + 5*E*_u_ + 16*T*_u_; it consists of six Raman active modes (*A*_g_, *E*_1g_, *E*_2g_, *T*_1g_, *T*_2g_, *T*_3g_) and three IR-active modes (*T*_1u_, *T*_2u_, *T*_3u_), and *A*_u_ and *E*_u_ represent inactive modes.^52^ The allowed number of Raman representations for the Wyckoff positions 24d and 48e are 8 and 15, respectively, but there is no Raman representation for the 8b site. This indicates indium atom in the 8b site does not involve in vibrational activity. Raman spectroscopy has shown that the frequency spectrum has the strongest peak at 501 cm^–1^, corresponding to the Raman-active *T*_g_ mode Figure 8a. An experimental and theoretical study by Garcia–Domene et al. indicates the values of Raman active peaks at 148, 152, 204, 476, 565, and 590 cm^–1^ observed for a cubic bixbyite-type crystal structure.^53^ These peaks are also close to the Raman peaks at 142, 155, 205, 476, 569, and 587 cm^–1^ of this study, listed in Table 6. The threefold degenerate *T*_g_ mode indicates in-plane symmetric stretch or bends of oxygen atoms with respect to a center of symmetry.^54^ The allowed number of IR representations for the Wyckoff positions 24d, 48e, and 8b are 5, 9, and 3 bands correspondingly. The strongest band in the spectrum occurs near 500 cm^–l^ and usually appears as broad. Many weaker absorption peaks are found (as shown in Figure 8b) at the low-frequency limit and higher frequencies.

**Table 6:** Raman and IR-Active Modes of Mechanically and Dynamically Stable In_2_O_3_ Polytypes

polytype	Raman active modes (in cm^–1^)	IR-active modes (in cm^–1^)
In_2_O_3_-*C*	^3^*T*_g_: 142, 155, 193, 256, 263, 405, 426, 476, 501, 569, .587, 643, 673, 763	^3^*T*_u_: 133, 164, 190, 201, 215, 267, 310, 412, 431, 471, 494, 517, 587, 658, 689, 725
	*A*_g_: 177, 420, 617, 739	
	^2^*E*_g_: 205, 417, 509, 730	
In_2_O_3_-*M*3	*A*_g_: 113, 119, 144, 202, 206, 281, 412, 454, 473, 581, 659, 678, 692, 780	*B*_u_: 101, 181, 220, 308, 395, 396, 446, 487, 574, 621, 681, 687, 694
	*B*_g_: 130, 131, 147, 196, 309, 311, 343, 378, 389, 525, 603, 653, 675, 769, 781	*A*_u_: 85, 119, 173, 198, 258, 284, 447, 487, 521, 533, 624, 667, 684, 739
In_2_O_3_-*M*4	*A*_g_: 102, 115, 147, 187, 228, 253, 270, 445, 455, 531, 564, 589, 654, 764, 822	*B*_u_: 157, 178, 197, 216, 239, 347, 466, 528, 565, 611, 681, 761, 771
	*B*_g_: 118, 157, 168, 182, 210, 231, 286, 449, 480, 554, 568, 603, 681, 815, 829	*A*_u_: 53, 91, 171, 211, 229, 275, 345, 427, 539, 565, 597, 655, 764, 795
In_2_O_3_-*O*1	*A*_g_: 89, 145, 198, 294, 336, 392, 496, 603, 696, 721	*B*_1u_: 105, 142, 240, 283, 413, 477, 571, 723, 760
	*B*_1g_: 82, 182, 383, 470, 639	*B*_2u_: 0, 135, 296, 435, 614
	*B*_2g_: 147, 185, 250, 277, 356, 544, 584, 611, 714, 753	*B*_3u_: 0, 74, 185, 213, 318, 393, 465, 552, 705, 759
	*B*_3g_: 178, 333, 461, 635	
In_2_O_3_-*O*3	*A*_g_: 136, 172, 205, 299, 371, 408, 537, 611, 650, 742	*B*_1_: 150, 234, 262, 336, 377, 428, 581, 678, 767
	*B*_1g_: 126, 166, 398, 473, 568	*B*_2u_: 135, 364, 448, 480
	*B*_2g_: 85, 218, 248, 290, 467, 524, 592, 619, 672, 743	*B*_3_: 135, 216, 242, 332, 380, 467, 651, 661, 738
	*B*_3g_: 142, 154, 442, 487, 536	

**Figure 9:**
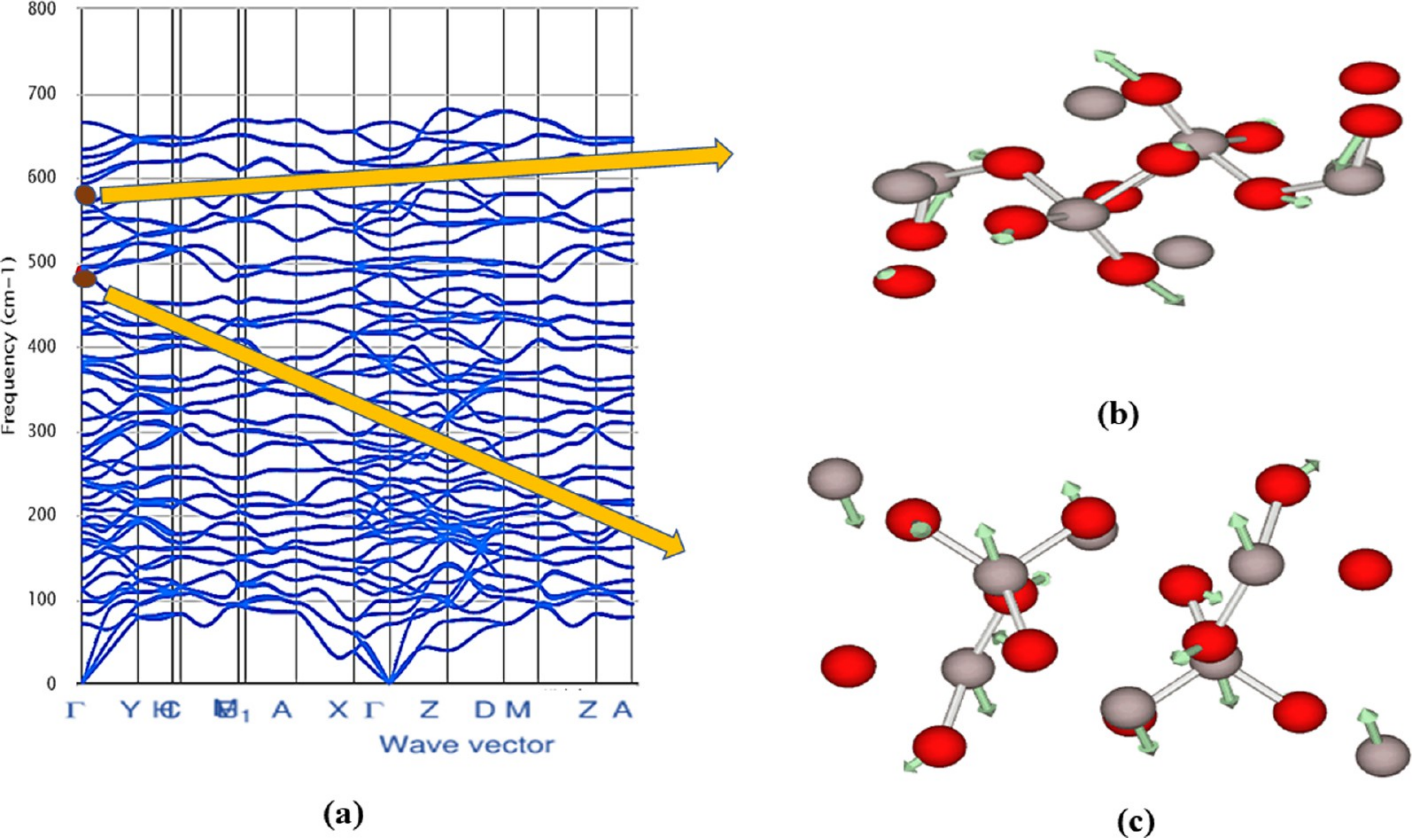
(a) Phonon dispersion curve of In_2_O_3_-*M*3 with (b) atomic displacements for the strongest Raman peak *A*_g_ (at 581 cm^–1^) and (c) for the strongest IR peak *A*_u_ (at 487 cm^–1^) vibration modes.

The symmetry of monoclinic In_2_O_3_ polytypes is defined by the point group *C*2*h*(2*/m*) and the irreducible representation of phonon modes is Γ = 10*A*_g_ + 5*B*_g_ + 4*A*_u_ + 8*B*_u_; it consists of two Raman active modes (*A*_g_, *B*_g_) and two IR-active modes (*A*_u_, *B*_u_).^55^ Raman spectroscopy of In_2_O_3_-*M*3 and In_2_O_3_-*M*4 has shown that the frequency spectrum with the strongest peak at 581, and 766 cm^–1^, respectively, corresponds to the Raman-active *A*_g_ mode. The nondegenerate mode *A*_g_ represents out-of-plane vibrations of oxygen atoms and they can be symmetric stretch or bend with respect to the principal axis of symmetry.^54^ A number of strong peaks are observed in the range of 700–900 cm^–l^. These are too high in frequency to be transverse optical modes. The allowed number of IR representations for the Wyckoff position 4e is 6 bands. The strongest absorption peak in the spectrum occurs at 487 cm^–l^ for In_2_O_3_-*M*3, which corresponds to the IR-active *A*_u_ mode and the atomic displacements for the strongest peak of Raman and IR vibrations are depicted in Figure 9. For In_2_O_3_-*M*4, the strongest absorption peak occurs at 565 cm^–l^ concerning IR *B*_u_ mode.

**Figure 10:**
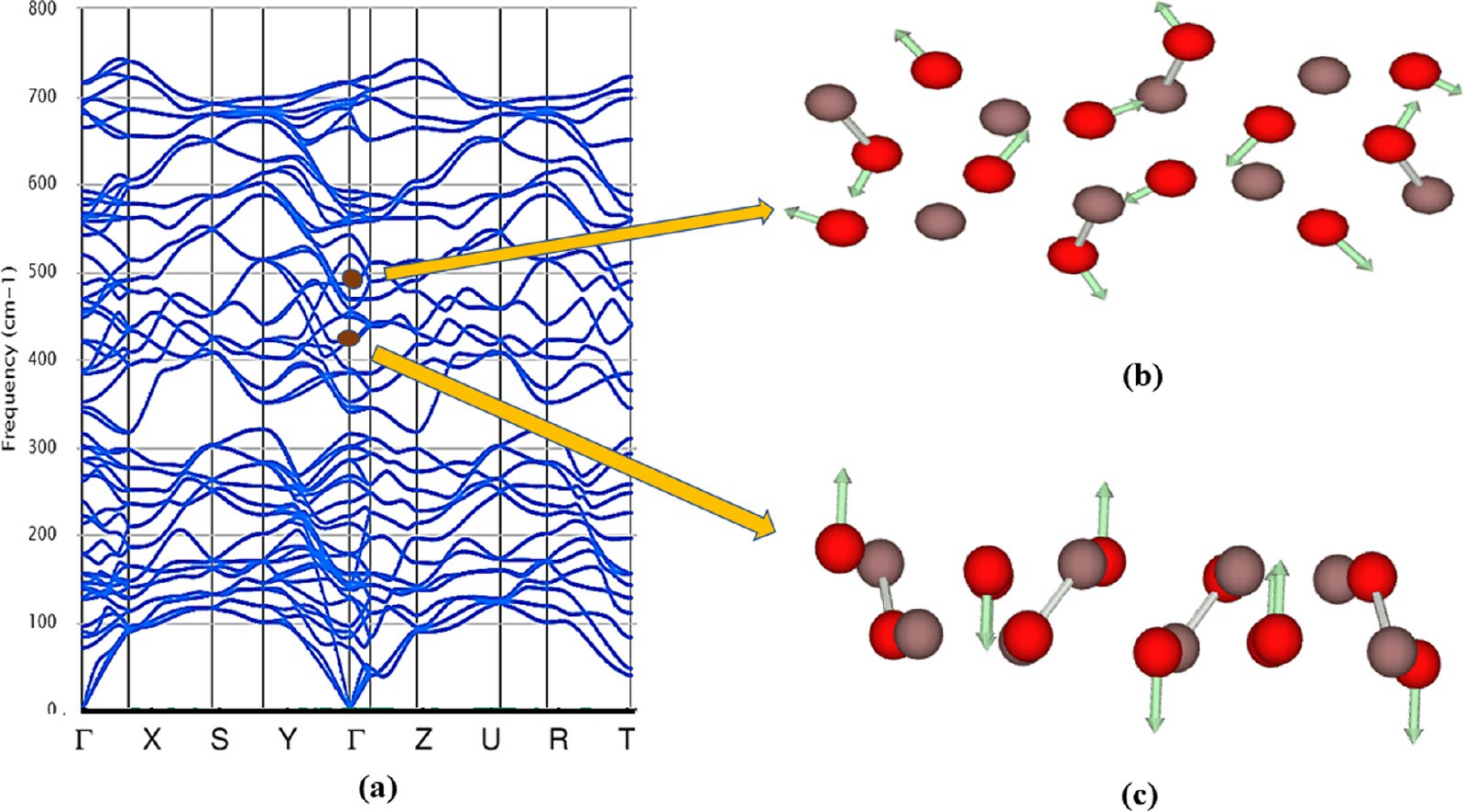
(a) Phonon dispersion curve of In_2_O_3_-*O*1 with (b) atomic displacements for the strongest Raman peak *A*_g_ (at 496 cm^–1^) and (c) for the strongest IR peak *B*_2u_ (at 435 cm^–1^) vibration modes.

The symmetry of orthorhombic In_2_O_3_ polytypes is expressed by the *D*2*h*(*mmm*) point group and the irreducible representation is Γ = 12*A*_g_ + 6*B*_1g_ + 12*B*_2g_ + 6*B*_3g_ + 11*B*_1u_ + 5*B*_2u_ + 11*B*_3u_; it consists of four Raman active modes (*A*_g_, *B*_1g_, *B*_2g_, *B*_3g_) and three IR-active modes (*B*_1u_, *B*_2u_, *B*_3u_).^56^ Raman spectroscopy of In_2_O_3_-*O*1 and In_2_O_3_-*O*3 has shown that the frequency spectrum with the strongest peak at 496, and 611 cm^–1^, respectively, corresponds to the Raman-active *A*_g_ mode. The nondegenerate mode *A*_g_ represents out-of-plane vibrations and they can be symmetric stretch or bend with respect to the principal axis of symmetry of the In_2_O_3_ molecule.^54^ The allowed number of IR representations for the Wyckoff position 4c is 5 bands. The strongest absorption peak in the spectrum occurs near 450 cm^–l^ for both In_2_O_3_-*O*1 and In_2_O_3_-*O*3 with respect to the IR-active *B*_2u_ mode. These vibrational modes are associated with rotational and the in-plane bending or stretching vibrations of oxygen atoms. For the polytype In_2_O_3_-*O*1, the atomic displacements for the strongest peak of Raman and IR vibrations are depicted in Figure 10.

## 3 Conclusions

Twelve In_2_O_3_ polytypes including eight new polytypes are projected and the relative stability is studied for the first time using DFT. The atomic equilibrium geometries are optimized and resultant lattice constants projected by Vienna ab initio simulation package (VASP) are promising with the available experimental values, which indicates the reliability of the present results. The energy–volume curve is evaluated, allowing for full relaxation at each volume and the data are fitted to the Birch–Murnaghan equation of state. Polytypes have their minimum energy between −27.9 and −26.5 eV/f.u., with a range of equilibrium volume between 60 and 145 Å^3^/f.u. The possible pressure-induced phase transitions are found from the pressure versus Gibbs free energy plot. At ambient conditions, In_2_O_3_ stabilizes in the In_2_O_3_-*C* phase. At higher pressure, In_2_O_3_-*C* is transformed to In_2_O_3_-*O*4 at 10.1 GPa, In_2_O_3_-*O*4 is transformed to In_2_O_3_-*O*2 at 12.9 GPa, and In_2_O_3_-*O*2 is transformed to In_2_O_3_-*O*3 at 41.7 GPa and agrees with the available reported results. From the electronic band structure (HSE-06 level) calculation, the bandgap (direct and indirect) values were found. The In_2_O_3_-*M*4 has the lowest energy band gap (1.5 eV) and two orthorhombic polytypes In_2_O_3_-*O*2 and In_2_O_3_-*O*4 have the highest band gap (3.94 eV) compared to others. The lower In_2_O_3_ conduction-band states are of oxygen 2p character, and the higher valence band states are of indium 5p character. Electronic band structure studies show the semiconductor characteristics of In_2_O_3_ polytypes. The bandgap range of all monoclinic In_2_O_3_ polytypes (1.2–1.97 eV) makes them viable for photovoltaic and photocatalytic applications. Other polytypes are suitable for photocatalytic and photovoltaic gas sensors and chemical sensors as their band gap vary from 2.4 to 3.94 eV. Born stability criteria of the elastic constant are fulfilled by all In_2_O_3_ polytypes with space groups *P*6_1_, *Ia*3̅, *P*2_1_*/_C_*, *Pnma*, and *Pbca*. It is observed that In_2_O_3_-*O*1 and In_2_O_3_-*O*3 have a more covalent nature and are stiffer than the remaining polytypes as their Young’s modulus value (*E* > 250 GPa) is very much higher than others. Likewise, the smaller value of *E* (<50 GPa) shows that In_2_O_3_-*H* and In_2_O_3_-*T*1 cannot resist huge tensile stress. Pugh’s ratio (*B*/*G*) and Poisson’s ratio confirm that all mechanically stable polytypes are ductile for the selected range of strains. To find the dynamic stability status, phonon studies are carried out for all mechanically stable polytypes, Confirming that the following polytypes In_2_O_3_-*C*, In_2_O_3_-*M*3, In_2_O_3_-*M*4, In_2_O_3_-*O*1, and In_2_O_3_-*O*3 are dynamically stable as they display positive modes. From the phonon dispersion, we found that lower frequencies are dominated by the smaller oxygen atom while the higher frequencies are dominated by the heavier indium atom. Negative (imaginary) phonon frequencies of In_2_O_3_-*H*, In_2_O_3_-*M*2, and In_2_O_3_-*O*4 indicate instability, causing the entire lattice to experience structural changes. The thermal parameters are measured and recorded, including heat capacity, free energy, and entropy. The hardness of In_2_O_3_-*M*3, In_2_O_3_-*M*4, and In_2_O_3_-*O*3 is confirmed by the high value of Debye temperature. Raman-IR studies on the most stable polytypes are done and listed. A thorough theoretical understanding of the stability study concludes that four new polytypes with space group (*P*2_1_*/c* and *Pnma*) comply with all the stability criteria other than experimentally proven cubic polytype In_2_O_3_-*C* (*Ia*3̅) and can be readily synthesized, and further experimental validation is essential.

## 4 Computational Methodology

By using DFT, the calculation of geometrical optimization and the electronic structure of In_2_O_3_ polytypes is supported using the code of VASP.^57^ The interaction between core and valence electrons of both indium (In: [Kr] 4d^10^ 5s^2^ 5p^1^) and oxygen (O: 1s^2^ 2s^2^ 2p^4^) is described employing the projected augmented-wave method.^58^ The In −4d, −5s, and −5p, O −2s and −2p electrons have been considered as the valence electrons. The GGA parameterized by Perdew, Burke, and Ernzerhof was utilized as an exchange-correlation functional.^59^ This relaxes the norm-conserving criteria and produces a smooth and computationally efficient pseudopotential without affecting the accuracy to a significant extent. The initial structures are taken as described in the Introduction section. For structural optimization calculation, the atomic positions, cell volume, and shape have been relaxed by force and by stress minimization.^60,61^ The total energy (*E*) as a function of the cell volume (*V*) is calculated using the optimized crystal structure information as an input. The magnitude of the equilibrium volume corresponds to the minimum (*E*_min_) of the total energy. The Monkhorst–Pack grid scheme is used to conduct the **k**-point sampling for the polytypes within the Brillouin zone.^46^ In order to provide a suitable level of energy convergence during cell volume computations, optimization is reached at the 550 eV energy cut-off value. A three-dimensional visualization program VESTA is used to visualize the volumetric data, calculated for the equilibrium structures.^62^ Band structures are obtained by the GGA and Heyd–Scuseria–Ernzerhof (HSE-06) screened hybrid functional.^63^ The elastic constant is computed by the finite strain method using post preprocessing tool VASPKIT.^64^ We use the supercell approach to accomplish our phonon computations.^65^ Supercell force constants are prepared, and phonon frequencies and phonon density of states are computed using the PHONOPY.^66^ Raman and IR spectra are produced from density functional perturbation theory as employed in the CASTEP package.^67^ We used the optimized structures with the same **k**-point mesh as the input for the CASTEP computation.

## Supporting Information Available

List of structure types involved in the selection of low-energy In_2_O_3_ polytypes, phonon dispersion relation, and spatial-dependent mechanical properties of the polytypes (PDF)
