# Supplementary material for: Structural, Electronic Properties, and Relative Stability Studies of Low-Energy Indium Oxide Polytypes Using First-Principles Calculations
Source: ACS Omega. 2023 Mar 30;8(14):12928–43. doi: 10.1021/acsomega.3c00105 (PMC10099427; doi:10.1021/acsomega.3c00105)
Supplement: Supplementary file 1 — ao3c00105_si_001.pdf [file ao3c00105_si_001.pdf]

Supplementary information of "Structural, electronic properties and relative stability studies of low-energy indium oxide polytypes using first-principles calculations"

Arthi Devamanoharan[a], Vasu Veerapandy[a] and Ponniah Vajeeston[b*]

[a] *Department of Computational Physics, School of Physics, Madurai Kamaraj University, Madurai 625021, India.*

[b] *Department of Chemistry, Center for Materials Science and Nanotechnology, University of Oslo, Oslo 0371, Norway.*

1. Selection of low-energy In_2O_3 polytypes

Involved input structure types in the selection of In_2O_3 polytypes are listed in Table S1.

Table S1: List of structure types involved in the selection of low-energy In_2O_3 polytypes

ICSD-15072-14	ICSD-1264-36	ICSD-9206-61	ICSD-65184-69	mp-1105699 In_2O_3	mp-22216_In_2S_3
ICSD-16021-144	ICSD-1376-169	ICSD-102092-123	ICSD-66362-19	mp-1194571_ In_2O_3	mp-22598_ In_2O_3
ICSD-26477-220	ICSD-1462-167	ICSD-10426-167	ICSD-73453-11	mp-2254_ Al_2O_3	mp-29502_Al_2Te_3
ICSD-31282-14	ICSD-15344-60	ICSD-33960-220	ICSD-73558-11	mp-32570_ Al_2O_3	mp-672673_In_2Se_3
ICSD-34291-12	ICSD-168808-115	ICSD-34243-12-8f	ICSD-74348-166	mp-32591_ Al_2O_3	mp-684676_Al_2S_3
ICSD-34685-36	ICSD-17008-160	ICSD-41017-2	ICSD-8014-43	mp-612740_In_2Se_3	mp-684944_In_2S_3
ICSD-4108-14	ICSD-170218-71	ICSD-419-62	ICSD-87415-191	mp-622511_In_2Te_3	mp-1017565_In_2Se_3
ICSD-58559-139	ICSD-1944-227	ICSD-43184-12	ICSD-9646-167	mp-638765_Al_2O_3	mp-1068510_In_2Te_3
ICSD-59605-88	ICSD-2033-56	ICSD-4478-160	mp-1105681 In_2O_3	mp-642363_ Al_2O_3	mp-1223831_In_2S_3
ICSD-61089-14	ICSD-2084-166	ICSD-488-9	mp-759943_Al_2O_3	mp-684990_ Al_2O_3	mp-1224450_In_2Te_3
ICSD-72292-11	ICSD-26864-189	ICSD-56771-164	mp-782035_Al_2O_3	mp-685036_ Al_2O_3	mp-19907_In_2Se_3
ICSD-78765-14	ICSD-26865-5	ICSD-57951-166	mp-985587_Al_2O_3	mp-752826_ Al_2O_3	mp-20830_In_2Se_3
ICSD-8056-12	ICSD-26867-65	ICSD-6095-167	mp-7048_Al_2O_3	mp-754531_ Al_2O_3	mp-22323_ In_2O_3
ICSD-9090-61	ICSD-30981-12	ICSD-621706-12	mp-754401_Al_2O_3	mp-755066_ Al_2O_3	mp-644741_ In_2O_3
ICSD-9091-206	ICSD-31750-164	ICSD-6286-15	mp-754624_Al_2O_3	mp-755175_ Al_2O_3	mp-673633_In_2S_3

2. Phonon dispersion

The phonon dispersion relation of the rest of the polymorphs were given in Figure S1.

* Corresponding Author

E-mail address: vajeeston.ponniah@kjemi.uio.no

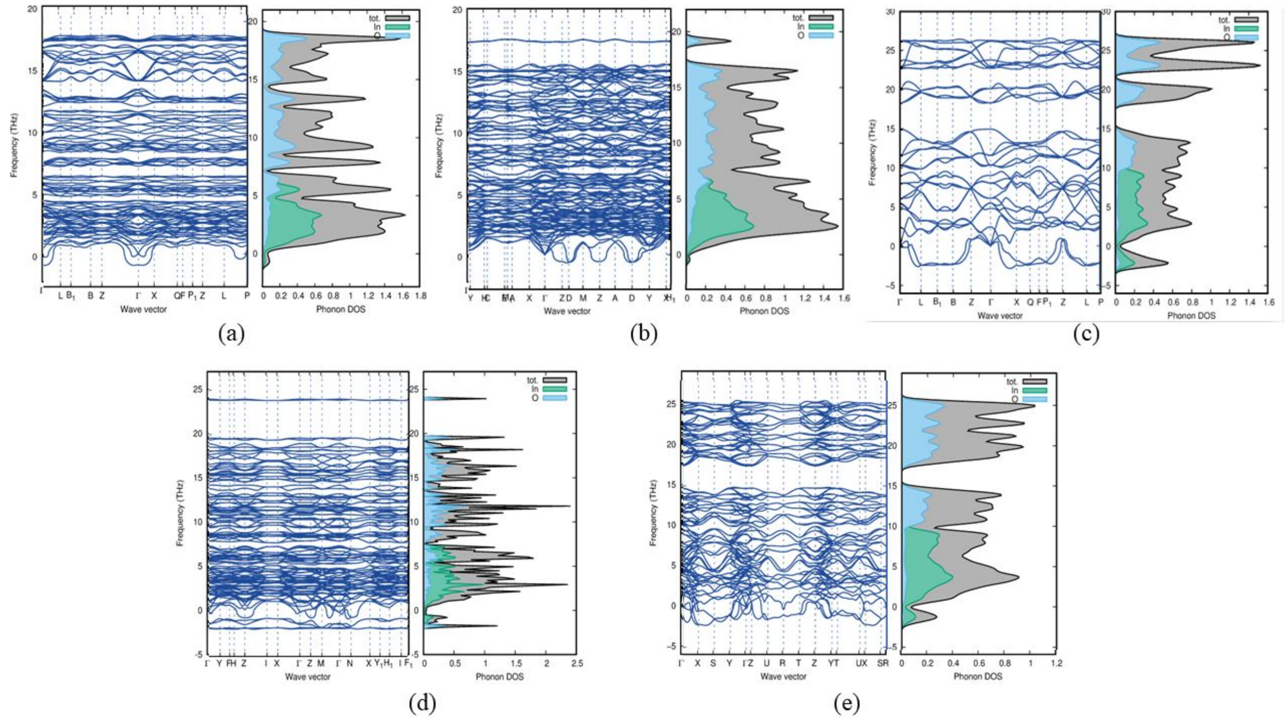

Figure S1: Phonon dispersion curve with PhDOS of mechanically stable In_2O_3 polytypes (a)In_2O_3-H (b)In_2O_3-*M1* (c) In_2O_3-*T1* (d) In_2O_3-*O2* and (d) In_2O_3-*O4* displays imaginary modes

3. Mechanical properties

3D spatial representations of Young's modulus, shear modulus and Poisson's ratio of all mechanically stable polytypes other than In_2O_3-*M2* are depicted in Figure S2, Figure S3, Figure S4 correspondingly.

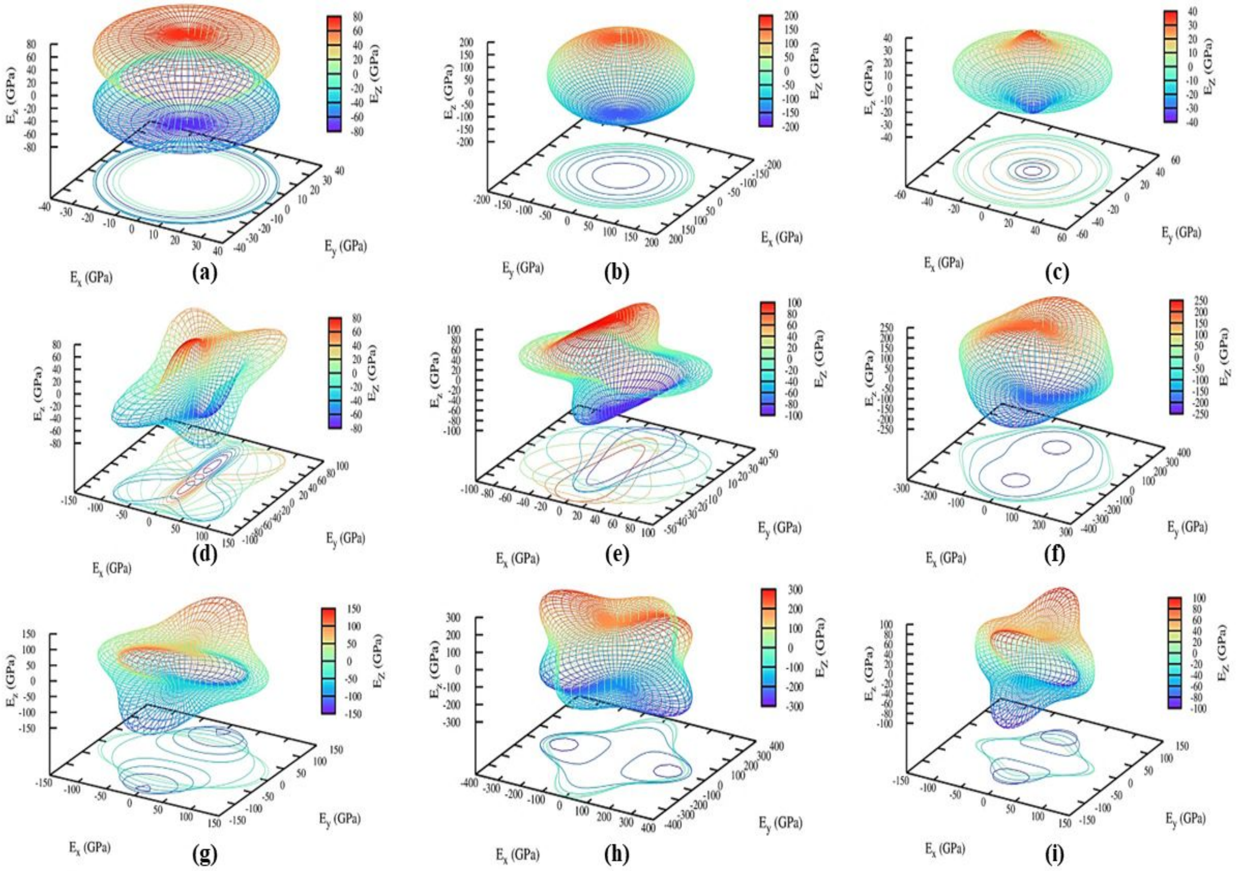

Figure S2 : 3D spatial dependence of Young's modulus of mechanically stable polytypes (a) In_2O_3-*H* (b) In_2O_3-*C* c) In_2O_3-*T1* (d) In_2O_3-*M1* (e) In_2O_3-*M3* (f) In_2O_3-*O1* (g) In_2O_3-*O2* (h) In_2O_3-*O3* and (i) In_2O_3-*O4*

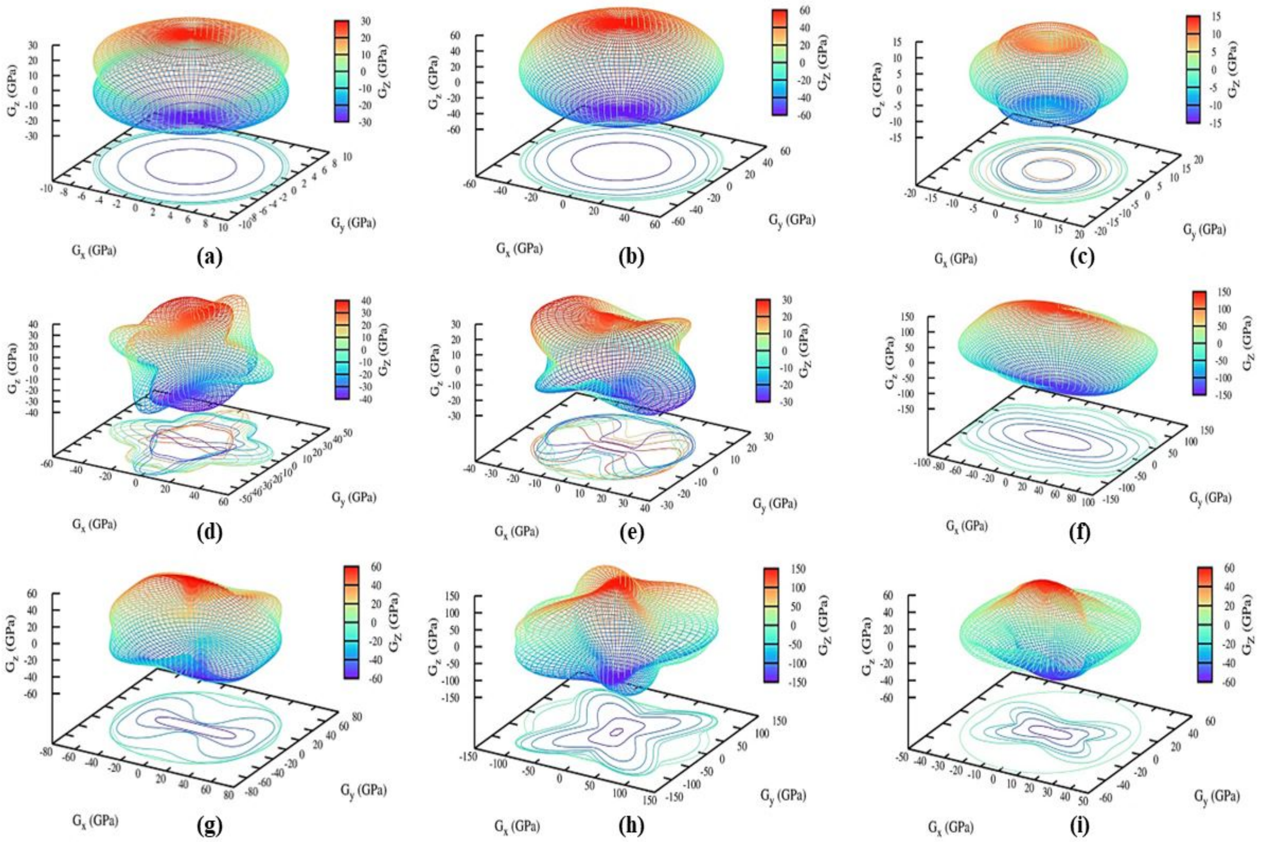

Figure S3 :3D spatial dependence of shear modulus of mechanically stable polytypes (a) In_2O_3-*H* (b) In_2O_3-*C* (c) In_2O_3-*T1* (d) In_2O_3-*M1* (e) In_2O_3-*M3* (f) In_2O_3-*O1* (g) In_2O_3-*O2* (h) In_2O_3-*O3* and (i) In_2O_3-*O4*

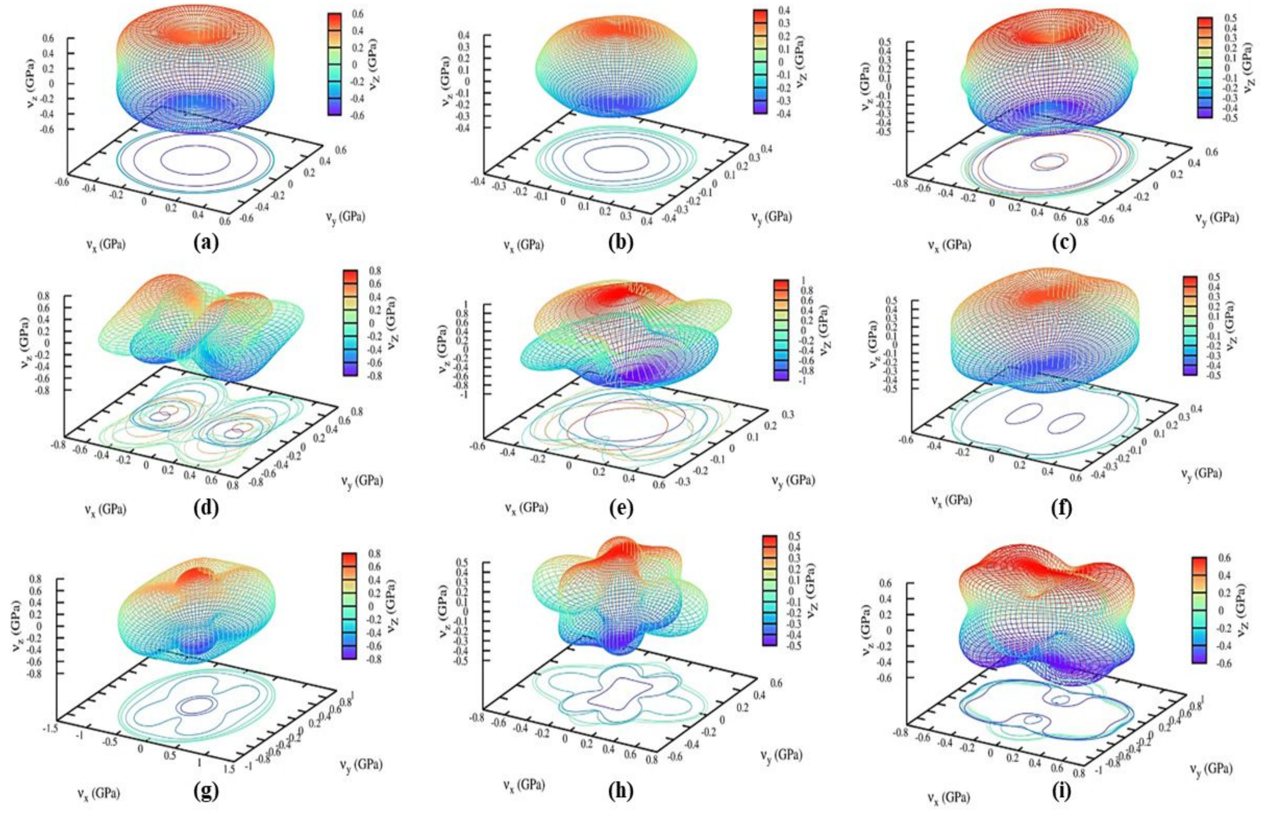

Figure S4: 3D spatial dependence of Poisson's ratio of mechanically stable polytypes (a) In_2O_3-*H* (b) In_2O_3-*C* (c) In_2O_3-*T1* (d) In_2O_3-*M1* (e) In_2O_3-*M3* (f) In_2O_3-*O1* (g) In_2O_3-*O2* (h) In_2O_3-*O3* and (i) In_2O_3-*O4*
